# EFSA’s toxicological assessment of aspartame: was it even-handedly trying to identify possible unreliable positives and unreliable negatives?

**DOI:** 10.1186/s13690-019-0355-z

**Published:** 2019-07-15

**Authors:** Erik Paul Millstone, Elisabeth Dawson

**Affiliations:** Science Policy Research Unit, University of Sussex, Brighton, BN1 9SL England

**Keywords:** Aspartame, EFSA, Appraisal, Asymmetry, Public health

## Abstract

**Background:**

A detailed appraisal is provided of the most recent (December 2013) assessment of the safety and/or toxicity of the artificial sweetener aspartame by the European Food Safety Authority’s Panel on Food Additives and Nutrient Sources Added to Food. That appraisal is prefaced with a contextualising chronological account drawn from a documentary archive of the key highlights of the antecedent scientific and policy debates concerning this sweetener from the early 1970s onwards. The appraisal focuses specifically on Section 3.2 of the panel’s review, which is headed ‘Toxicological data of aspartame’.

**Methods:**

The methodology of the appraisal focusses on the extent to which the panel was symmetrically alert to possible false positives and false negatives, which in toxicological terms denote misleading indications of possible toxicity or misleading indications of safety. The methodology involved identifying and tabulating the prima facie indications of each of 154 empirical studies, and then comparing them with the way in which the panel chose to interpret the studies’ findings, by focussing primarily on whether the panel deemed those studies to be reliable or unreliable. If the panel had been even-handed, the criteria for assessing reliability should have been the same for both putative positive and negative studies.

**Results:**

Eighty-one studies were identified that prima facie did not indicate any possible harm, and of those the panel deemed 62 to be reliable and 19 as unreliable. Seventy-three studies were identified that prima facie did indicate possible harm; of those the panel deemed all 73 to be unreliable; none were deemed reliable. A qualitative comparative review of the criteria of appraisal invoked by the panel to judge the reliability of putative negative and positive studies is also provided.

**Conclusion:**

The quantitative result indicate that the panel’s appraisal of the available studies was asymmetrically more alert to putative false positives than to possible false negatives. The qualitative analysis shows that very demanding criteria were used to judge putative positive studies, while far more lax and forgiving criteria were applied to putative negative studies.

**Discussion:**

That quantitative and qualitative patterns are very problematic for a body supposed to prioritise the protection of public health. Given the shortcomings of EFSA’s risk assessment of aspartame, and the shortcomings of all previous official toxicological risk assessments of aspartame, it would be premature to conclude that it is acceptably safe. They also imply that the manner in which EFSA panels operate needs to be scrutinised and reformed.

**Electronic supplementary material:**

The online version of this article (10.1186/s13690-019-0355-z) contains supplementary material, which is available to authorized users.

## Background

Amongst food additives, aspartame is one of the most controversial, especially in the USA, but also in the UK and the EU. The most recent official attempt to settle the controversy was provided by the European Food Safety Authority’s (or EFSA) Panel on Food Additives and Nutrient Sources added to Food (or ANS) in December 2013 [1]. The ANS Panel: “… concluded that aspartame was not of safety concern at the current aspartame exposure estimates or at the ADI [acceptable daily intake] of 40 mg/kg bw/day” [2]. An ADI is a level of consumption officially deemed to be acceptably safe.

In the context of a set of proposals to transform the European Food Safety Authority into an ‘Open EFSA’, a 2014 discussion paper from the EFSA Board highlighted some of the intended benefits from the openness and transparency to which it claimed to aspire. The text stated:

“The proactive and committed adherence to openness and transparency values by the Authority facilitates an informed debate, both among experts and the public, on scientific issues within EFSA’s remit. Thereby this represents a prerequisite for constructive and informed dialogue between the agency and any interested organisation or individual.” [3].

This paper is in part intended to contribute to a constructive and informed dialogue with EFSA and with other stakeholders. This paper will highlight the limited extent to which the ANS panel’s risk assessment of the artificial sweetener aspartame was in practice transparent. EFSA also stipulated that its risk assessments should be ‘reproducible’ [4], correspondingly this paper will also specify the conditions under which it might be reproducible, in terms of the assumptions that would need to be made.

This paper has two main sections. The first provides a chronological account drawn from a documentary archive of the key highlights of the antecedent scientific and policy debates concerning the safety and/or toxicity of aspartame from the early 1970s onwards, while the second provides a critical review of the December 2013 ANS report and explains why it did not settle the controversy but rather contributed to it. The central question addressed in the second section asks whether the ANS panel’s review of toxicological evidence was symmetrically sceptical? In other words, did it even-handedly try to identify possible unreliable positives (ie studies indicating adverse effects, but unreliably) and unreliable negatives (ie studies not indicating adverse effects, though unreliably), or was it asymmetrically focused more on one than the other?

The answer that emerges from a detailed quantitative and qualitative scrutiny of the studies, and the ANS panel’s representations and interpretations of those studies, is that the panel frequently treated studies that provided no prima facie evidence of harm as if they were unproblematically reliable, even when they were very weak studies, yet discounted the results of every single one of 73 studies that indicated that aspartame could be harmful, deeming all those studies to be unreliable and/or insufficient, even though some were more powerful and sensitive than some of the seemingly negative studies that the panel deemed reliable.

Furthermore, the evidence shows that, if the benchmarks that the ANS panel used to evaluate the results of ‘negative’ studies had been consistently used to evaluate the results of ‘positive’ studies then the panel would have been obliged to conclude that there was sufficient evidence to indicate that aspartame is not acceptably safe. Correspondingly, if the benchmarks that the ANS panel used to evaluate the results of positive studies had been consistently used to evaluate the results of negative studies then the panel would have been obliged to conclude that there was insufficient evidence to conclude that aspartame is acceptably safe. Instead it said that aspartame is safe when consumed at currently estimated rates of consumption. This paper therefore questions the procedure followed by the ANS panel and its conclusion.

Given that animal experiments conducted to test, for example, the safety and/or efficacy of chemicals often generate findings that are equivocal and/or conflicting, considerable efforts have, in recent years, been devoted to developing suitable methods by which to provide systematic reviews of the diverse results of such studies to indicate the overall implications of multiple datasets. One promising example of such novel methods is known as SYRCLE, which is an adapted version of the Cochrane Risk of Bias tool [5]. An alternative approach is known as CAMARADES, which is an acronym for ‘Collaborative Approach to Meta-Analysis and Review of Animal Data from Experimental Studies’ [6]. Both SYRCLE and CAMARADES focus on trying to identify ‘risk of bias’ in animal studies, though their applicability does not extend to epidemiological or clinical studies, though Cochrane Collaboration .guidelines can be applied to those studies. Given that this paper is a review of a central part of one single EFSA document, those approaches are not applicable in this context. However, as and when future official public policy reviews of the safety and/or toxicity of aspartame and other chemical additives, contaminants and nutrients will be conducted, such systematic approaches should be adopted, and their selection and application explained and justified.

### Section 1

#### Regulatory, scientific and corporate context

A good case can be made for the claim that no industrial food additive has been more controversial than aspartame [7]. G D Searle, a US pharmaceutical company, first petitioned the US Food & Drug Administration (FDA) for permission to market aspartame in 1973; in 1974 the FDA announced its intention to grant permission for its use in dry goods, such as table-top sweeteners [8]. Before that decision could be implemented, objections were raised by independent scientists alleging that aspartame could cause mental retardation, brain lesions and neuroendocrine disorders [9]. Before those issues were resolved, further objections were raised focussing on documentary and interview evidence indicating that Searle, and its sub-contractor Hazleton, had failed to conduct properly at least 15 toxicology tests, and that their subsequent reports had been misleading [10].

The key allegation was that the conduct of those studies was seriously incompetent, and that when Searle managers recognised the failures that had taken place they mis-reported the studies to conceal those failures, portraying the studies as if they had been conducted competently and reported accurately. The FDA could not simply discard or discount the evidence it had collected, which revealed that it had initially been misled by unreliable studies and reports, so instead it was discarded and discounted through several separate processes.

The FDA arranged for the 15 problematic studies to be reviewed, but in two separate sets and by different institutions. The FDA’s Bureau of Foods convened 5 of its staff into a Task Force and assigned it to review just 3 of those 15 studies [11]. The job of reviewing the other 12 studies was assigned to an organisation called the US Universities Association for Research and Evaluation in Pathology (UAREP). The FDA negotiated an agreement with G D Searle under which Searle would pay the costs of the UAREP work, in exchange for which the FDA agreed that Searle would contribute to setting the UAREP review’s terms of reference [12]. Dr. Adrian Gross, who was then an FDA pathologist, and who first uncovered the problems with Searle’s laboratory work and reporting, argued in 1976 that the members of the UAREP team were not appropriately qualified to conduct the kind of investigation that was required, and consequently that their eventual conclusions could not be considered to be reliable or definitive [13]. Gross had been instrumental in uncovering the shortcomings in Searle’ tests on aspartame as well as on two pharmaceutical products (flagyl - an antibiotic, and aldactone - a diuretic) [14]. Gross persistently criticised all 15 of the studies, ie both those reviewed by the Bureau of Foods Taskforce and those reviewed by the UAREP.

Both the Bureau of Foods Task Force and the UAREP subsequently issued reports containing reassuring conclusions, but in both cases they only contrived to reach those conclusions because their terms of reference had been set particularly narrowly and in ways that failed to address the critical shortcomings of the studies. Both teams focussed their attention on characterising and comparing the remaining parts of the documentary records of those studies alongside the detectable features of laboratory samples, including samples of animals’ tissues on glass slides, but without examining the prior processes that had resulted in those documents being written and the samples remaining available [15]. The report of the UAREP never explicitly said that the reviewers took Searle’s documentary and laboratory evidence at face value, but that in practice was what happened. On a few occasions, the UAREP highlighted omissions from documents, and inconsistencies between them, but otherwise treated them as if they were unproblematically reliable. The UAREP was not provided with evidence of the incompetence of some of the laboratory staff or the fictional aspects of some of the documentation. They took the documents and the pathology slides at face value, and checked the arithmetic. Although UAREP noted “… a substantial number of minor and inconsequential discrepancies …” during its review, it found “… few, if any, discrepancies which would produce a change of greater than five percent in the final numerical data being compared” [16].

#### The FDA bureau of food task force report

The conclusion of the Bureau of Foods Task Force stated that while the three tests had not been properly conducted, and although there were marked differences between raw data and the summaries submitted in the petition to the FDA, those differences: “…were not of such a magnitude that they would significantly alter the conclusions of the studies” [17]. The three studies reviewed by the Bureau of Food Task Force were listed using Searle’s numbering system as: E5, E-89 and E-77/78. The last of those three studies has been omitted from our quantitative analysis below because it was a study of the toxicity of diketopiperazine (or DKP) which is one of aspartame’s breakdown products, rather than a study of aspartame itself. It was however included in Section 7.2.4.1 of the ANS panel’s report, where it was interpreted by the panel as a reliable negative.

The Task Force had considerable difficulty in evaluating the studies, in part because in some cases there were no raw data with which to compare the reported results. In other cases, it was impossible to determine which the real raw results were and which were subsequent revisions or summaries. In some contexts, the Task Force had to rely on information and assumptions provided by Searle employees who had not been involved in the original work. At worst, it was impossible to identify the occasion on which a particular animal had died. For example, the report said in relation to E78/79, a study of the toxicity of DKP: “Observation records indicated that animal A23LM was alive at week 88, dead from week 92 through week 104, alive at week 108, and dead at week 112” [18]. Most scientists do not believe in reincarnation, and we should not expect that the FDA or the ANS panel to do so either.

When reviewing the test (E78/79) on DKP, the report listed no fewer than 52 major discrepancies in the Searle submission [19]. One of the central problems concerned the quantities of DKP supposedly consumed by the rats. The FDA investigators found no fewer than 3 separate documents with different specifications for the content and the purity of the test substance, and they were unable to establish precisely which specification, if any, was correct. It was impossible to reconcile the quantity of the chemical requisitioned from stores with the quantities supposedly fed to the animals. There were questions raised as to the extent to which the DKP was uniformly incorporated into the animals’ food. There is clear evidence to show that the test substance was not properly ground, and inadequately mixed, so that it might have been possible for the animals to avoid the DKP while eating their food [20].

Ten years later, at a November 1987 hearing of a US Senate Committee hearing Dr. Jacqueline Verrett, an FDA toxicologist who had been a member of the Bureau of Foods Task Force, explained that the three studies it had examined were “… woefully inadequate …” (p 387) and that: “Almost any single one of these aberrations would suffice to negate a study designed to assess the safety of a food additive, and most certainly, a combination of many such improper practices would, since the results are bound to be compromised. It is unthinkable that any reputable toxicologist giving a completely objective evaluation of this data resulting from such a study could conclude anything other than that the study was uninterpretable and worthless and should be repeated.” [21]. Nonetheless, the ANS panel included E5 in Section 3.2 of its December 2013 review, but deemed it an unreliable positive; though it deemed E89 to be a reliable negative.

When asked to explain the contrast between the 1977 report of the Bureau of Foods Task Force and her subsequent statements to the 1987 Senate committee, Jacqueline Verrett explained that the Task Force members were: “… limited in what we could actually conclude about the studies. We were not allowed to comment on the validity of any study. It was an explicit instruction based on administrative rather than scientific considerations. We were supposed to figure out what the conclusions would have been if the studies had been fully and correctly reported. We were obliged to ignore the protocols and the non-homogeneity of the DKP … Some animals did reject the DKP. Searle initially said that it may not have been fully mixed but that that did not matter, they later said that it had been fully mixed. We were not allowed to consider those issues by the Bureau of Foods administrator … We were ham-strung in being able to comment. The fact is that the studies should not have been considered at all, and that was the position from the beginning.” [22].

#### The report of the UAREP

In 1978, the UAREP delivered its 1062-page report, which concluded that the 12 studies they had audited were “authentic” [23]. Gross subsequently told the November 1987 US Senate committee hearing that:

“… no amount of additional examinations of pathology material such as undertaken by the UAREP … [or] … new additional statistical analyses … and no judgmental evaluations or interpretations of any data arising from those studies can in any way rectify the basic problem …: in the absence of reasonable expectation that the experimental animals were administered the correct dosages of the test agent, any observational data carried out on those animals must be regarded as questionable or flawed. This is to say nothing of all the myriad of other problems involving the competence of those conducting such studies, and the [lack of] care they exercised in their execution. Once a study is carried out and the test animals are disposed of, all that remains are the number of tiny bits of tissue preserved from their organs for microscopic examination and the written records of observations made by those who actually carried out that study. While the tissues themselves can be examined by others long after the remains of those animals no longer exist, the reliability of the written records has already been found to be unacceptable in a great variety of ways. … Once a study is compromised in its executions, it is beyond salvation by anyone. Even with respect to those small portions of tissue preserved for microscopic examination for an indefinite period of time after any study is completed there are serious problems … there is little if any assurance that such samples of tissues as were preserved actually originate from the specific animals said … to have been their source … Furthermore, due to the unacceptably high rate of post-mortem autolysis, a great many such tissues were not collected at all from the experimental animals.” [11].

The 12 studies for which the UAREP was responsible for reviewing were listed as: E-28, E-33 & 34, E-70, E-75, E-76, E-86, E-87, E-9, E-11, E-19, E-88 and E-90. Of those, E-76 was not included in the ANS panel’s discussion of the toxicity of aspartame, but it was included in relation to DKP. It is consequently omitted from our quantitative analysis below. When referring to the studies reviewed by the UAREP, Verrett said: “… the safety of aspartame and its breakdown products has still not been satisfactorily determined, since many of the flaws cited in these three studies [those reviewed by the FDA Task Force] were also present in all the other studies submitted by Searle [including those reviewed by UAREP]” [24].

Despite the fact that those two reviews had provided reassurances, several objectors remained dissatisfied, and furthermore a new complex set of objections to the safety of aspartame were introduced [25]. In an attempt to resolve the controversy once and for all, the FDA proposed the establishment of a so-called Public Board of Inquiry (or PBoI). This was a unique institution; the procedure had never previously been used, and in all probability will not be used again [26]. The PBoI first met in early 1980, and published its conclusions in October 1980 [27]. Two sets of issues were on its agenda. On one of the crucial questions its view was that aspartame consumption would not pose an increased risk of brain damage resulting in mental retardation, but on the other issue it concluded that the evidence available did not preclude the possibility that aspartame could induce brain tumours. Consequently, the Board recommended that aspartame should not be permitted for use, pending the results of further studies.

#### The FDA’s attempted follow-up

In 1976, the evidence of misconduct by Searle, in relation to two pharmaceutical products and aspartame, was sufficient to convince the FDA’s Chief Counsel (Richard Merrill) to instruct the Federal Attorney in Chicago to convene a Grand Jury to investigate: “… apparent violations of the Federal Food, Drug, and Cosmetic Act … and the False Reports to the Government Act … by G.D. Searle and Company and three of its responsible officers for their willful and knowing failure to make reports to the Food and Drug Administration required by the Act … and for concealing material facts and making false statements in reports of animal studies conducted to establish the safety of … Aspartame.” [28].

A team of investigators working for US Senator Metzenbaum subsequently gathered and then released a set of documents showing that soon after the Chicago Federal Attorney (Samuel Skinner) received Richard Merrill’s April 1976 letter, he was invited to join the board of the firms of lawyers (Sidley & Austin) then representing G D Searle; he accepted the invitation. The Searle dossier remained suspended until the next Federal Attorney (Thomas Sullivan) was appointed; he too was then invited to join the board of Sidley & Austin, and accepted the invitation [29]. Those processes served to delay legal proceedings until the interval, specified by the Statute of Limitations, had expired. Searle was therefore not prosecuted, but that did not establish the corporation’s innocence.

#### Informing EFSA

Documents providing detailed empirical support for the above account of the toxicological and regulatory history of aspartame were included in a dossier provided by Erik Millstone to the secretariat of the ANS panel in October 2011. The Secretariat had issued a public request for “… all necessary data (published, unpublished or newly generated) …” on 1st June 2011 [30]. Millstone responded by sending EFSA an annotated list of 30 relevant documents. The Head of the ANS Unit replied requesting by 4 November 2011 digitised copies of 27 of the documents [31]. 26 of the 27 documents were then fully digitised and dispatched to EFSA on a CD-ROM. The exception was the UAREP report, because at 1062 pages, only the Table of Contents was provided. In the event, the ANS panel’s reports of both January and December 2013 failed to mention that dossier, or most of the documents of which it was comprised, which was unjustified when judged by reference to both scientific and policy criteria. Copies of those documents are available at http://www.sussex.ac.uk/spru/research/projects/fcs.

#### Aspartame’s eventual approval

It was not until 1981, and the arrival of the Reagan administration, that the FDA permitted aspartame’s commercial use, restricting it initially to dry goods [32]. In 1983 the FDA approved its use in beverages, which became its major market for which it was marketed under the name ‘Nutrasweet’ [33]. The eventual approval process was seriously problematic. The FDA Commissioner’s decision was taken against the advice of FDA toxicologists and the Public Board of Inquiry [34]. Commissioner Hayes approved aspartame as one of his first decisions in that post, and was subsequently employed by the US National Soft Drinks Association [35].

When the Reagan administration assumed office in early 1981, Searle’s former CEO accepted the job as the new US ambassador to Beirut, in exchange for a promise that the administration would get aspartame onto the market [36]. The Reagan administration took office a short while after a terrorists group truck-bombed the US embassy in Beirut. Reagan appointed as his new ambassador to Beirut the former chief executive of GD Searle, Donald Rumsfeld.

Aspartame was deemed acceptably safe by the World Health Organisation and UN Food and Agriculture Organisation's Joint Expert Committee on Food Additives (JECFA) in 1980 and by the European Commission’s Scientific Committee for Food in 1985 [37]. Aspartame was also approved in the United Kingdom (UK), on advice from the Committee on Toxicology (CoT), the chair of which had his research indirectly funded by Searle [38]. All of those committees based their judgements on sets of studies that included those that Verrett had accurately characterised as the ‘woefully inadequate’. They were included and evaluated as if they were no more problematic than any other studies; their severe shortcoming were ignored or discounted, or maybe they were not drawn to the attention of the members of those committees. Several of the members of those committees were, moreover, acting as paid consultants to relevant food, beverage and chemical companies, in circumstances when declarations of conflicts of interest were then not required [39]. It would be naive to presume that undeclared conflicts of interest could not have influenced the judgements of those committee members.

#### Subsequent twentieth century developments

In October 1985 it emerged that Searle had been acquired by the large chemical company Monsanto, which had long been one of the major manufacturers of Saccharin [40]. Monsanto subsequently detached the aspartame business from the remainder of Searle’s operations and established the NutraSweet Company [41].

After aspartame had been approved the controversy shifted to discussions of reports from consumers of acute adverse effects [42]. The most common symptoms were (and are) neurological problems including severe headaches and blurred vision; thankfully reports of epileptic-type seizures, though serious, are rare. Such evidence has repeatedly been officially dismissed as ‘anecdotal’, though the sufferers often report that when consumption ceases so too do the symptoms. Moreover, when symptoms recur, the sufferers of those symptoms often discover that they had inadvertently consumed aspartame [43].

In the 1990s one key development was a paper by Olney et al. in the *Journal of Neuropathology and Experimental Neurology* suggesting that the introduction of aspartame into the USA may have resulted in a rapid increase in the incidence of a particularly aggressive type of brain tumour [44]. Their evidence was however officially discounted, despite providing convergent indications from animal studies, in vitro mutagenicity tests and human epidemiological data.

#### Twenty-first century debates

At the end of the twentieth century there were, consequently, good grounds for concluding that no one could be confident that aspartame was acceptably safe. In this century, the main toxicological contributions were provided by the Ramazzini Foundation’s rodent carcinogenicity studies [45].

The conventional protocols for long-term rodent feeding studies typically involve feeding a test compound to four dose-groups of animal, with 50 males and 50 females in each of low-dose, mid-dose and high-dose groups as well as a corresponding control group, making a total of 400 animals. The typical duration for carcinogenicity studies in rodents is 104 consecutive weeks [46], although the OCED has indicated that, while the duration will normally be 24 months for rodents, for specific strains of mice, 18 months may be more appropriate [47].

The first Ramazzini study on aspartame, published in 2005, used 1800 rats. Instead of testing the compound at three dose levels (plus a control group) they tested it at six dose levels plus controls. Instead of killing the rats at 2 years of age, the rats were allowed to live longer so that long-term effects could be studied. Subsequently, the Ramazzini Institute published data from a mouse study of aspartame, and a further rat study that included in utero exposure [48]. Gift et al. have argued that: “The protocols characteristic of RI [Ramazzini Institute] studies can cause interpretive challenges, but aspects of the RI design, including gestational exposure, life span observation, and larger numbers of animals and dose groups, may impart advantages that provide chemical risk assessors with valuable insights for the identification of chemical-related neoplasia not obtained from other bioassays” [49].

In these and many other ways, the Ramazzini study was more thorough, sensitive, reliable and relevant to human exposure than those conducted in accordance with conventional protocols. The authors reported in 2005 that their study: “… demonstrated for the first time that APM [aspartame] is a multipotent [ …] carcinogenic agent …” with dose-related tumour increases in both males and females [50]. In 2010 the Ramazzini team published the results of a study showing that aspartame induced tumours in the livers and lungs of male mice [51].

Several official bodies, including the US FDA, JECFA, the Scientific Committee on Food (SCF) of the European Commission and the UK’s CoT discounted those findings, complaining that the Ramazzini studies had not followed standard protocols. While the Ramazzini protocols were non-standard, their deviations from the standard, by using more animals in more dose groups and not ‘sacrificing’ them prematurely entailed that the Ramazzini studies provided greater sensitivity than could be obtained from a standard study. Keeping the animals until they die may not be common practice, but since European Union (EU) food safety policy legislation stipulates that “Assuring that the EU has the highest standards of food safety is a key policy priority …” [52] we might have expected that EFSA’s benchmark would be the protection of *all* consumers throughout their entire lives, rather than, for example, only until they reach retirement age. Those considerations imply that the Ramazzini protocol can be expected to provide a better model of the risks to the population of Europe than studies that ‘sacrifice’ the animals prematurely, however orthodox they might be. Premature sacrifice might well result in ‘unreliable negatives’, and unacknowledged ones at that.

One reason why the findings of the Ramazzini rat study had been officially discounted was because of relatively high rates of respiratory infections in the elderly rats [53]. However, as the rates of infection in the test groups were not significantly different from that in the control group, those infections could not explain the dose-related increase in tumours. Caldwell et al. convincingly rebutted the hypothesis that lymphomas and leukaemias were induced by infection. They noted, for example, that while respiratory infections frequently occur in old rats, and in most Ramazzini Institute rat bioassays, leukaemia and lymphoma were only reported in a few animals, namely 8 out of 112, implying that a link between the respiratory infections and those pathologies was improbable [54].

Another reason why the findings of the Ramazzini Institutes studies have been officially deemed not to be reliable has been because the tumour rates in treated animals were within the ranges reported for historical controls, even though they showed significantly higher rates that those in the concurrent controls. One reason why that criterion of interpretation is problematic was articulated by the WHO’s International Agency for Research on Cancer, which had emphasised in January 2006 that: “It is generally not appropriate to discount a tumour response that is significantly increased compared with concurrent controls by arguing that it falls within the range of historical controls ….” [55].

In 2013 the EFSA ANS panel, in line with other statutory risk assessors, discounted the Ramazzini findings as a set of unreliable positives, while accepting as reliable negatives, the evidence of studies that had many more, and far more serious, imperfections including those Verrett previously characterised as ‘woefully inadequate’. Inevitably, there were imperfections in the Ramazzini studies, but all studies are characterised by some imperfections. The ANS panel chose to treat many of the 15 earlier studies [56] (discussed above) as reliable, despite the fact that their imperfections were very substantially greater than those that characterised Ramazzini’s work.

Despite the efforts of EFSA, and a coalition of industrial and commercial stakeholders, to provide reassurances about the safety of aspartame, the accumulation of fresh evidence and public concerns provoked the European Parliament’s Public Health and Consumer Protection Committee to call on the Commission formally to instruct EFSA urgently to initiate a review of aspartame’s toxicity and safety, rather than keeping to a previously-set target-date of 31 Dec 2020 before doing so. In May 2011 the European Commission asked EFSA to re-evaluate the safety on aspartame (E951) as a food additive, and to do so by 31 July 2012 [57].

The ANS panel issued a 245-page ‘draft report’ in January 2013, and requested comments by 15 February 2013 [58]. The abstract of that document stated that the Panel had: “… concluded that there were no safety concerns at the current ADI of 40 mg/kg bw/day. Therefore, there was no reason to revise the ADI for aspartame.” [59] The panel’s draft was problematic in numerous respects; it failed to address the key issues concerning the unreliability of the 15 studies, namely those that had previously been reviewed by the FDA Bureau of Foods Task Force (ie E5, E-89 and E-77/78) and those previously reviewed by the UAREP (ie E-28, E-33 & 34, E-70, E-75, E-76, E-86, E-87, E-9, E-11, E-19, E-88 and E-90), on which Erik Millstone had provided the ANS panel’s secretariat with detailed documentary evidence. All of those 15 studies were cited in the ANS panel’s report, and the only relevant comment on them cited the UAREP’s document, but failed to refer to Gross’s devastating critique of the relevance and reliability of the UAREP review [60].

The way in which the studies, which had been included, had been interpreted appeared consistently inconsistent [61]. The ANS panel had portrayed most of the studies that did not indicate any possible harm, except at dose levels above the nominal ‘no-observed-adverse effect-level’ (or NOAEL) of 4000 mg of aspartame per kilogramme of the body weight of the test-species per day (ie mg/kg bw/day) as unproblematically reliable, while portraying each and every one of the studies indicating possible harm as unreliable and/or inconclusive, even though many of the studies providing positive evidence of toxicity were far more sensitive and rigorously conducted and reported than some of the apparently negative studies [62].

EFSA issued a ‘final report’ on 10 Dec 2013. It culminated with: “Overall, the Panel concluded from the present assessment of aspartame that there were no safety concerns at the current ADI of 40 mg/kg bw/day. Therefore, there was no reason to revise the ADI for aspartame.” [63].

Millstone responded on 14 December with a 3-page critique arguing that the panel had consistently treated a very large majority of negative studies as reliable, while discounting each and every one of the studies providing evidence of harm as unreliable [64]. In response, EFSA’s Head of Regulated Products and senior colleagues held a video-conference on 14 April 2014 with Millstone. On 14 November 2014 EFSA’s Head of Regulated Products wrote to Millstone, referring to a full list of studies. The letter reported that an internal review had concluded that:

“ … we found that the number of studies not indicating harm considered in the [ANS] opinion on aspartame as unreliable was substantially higher (35%) than the number claimed in your letter (20%). Likewise our analysis showed that the number of studies indicating harm by aspartame and treated by the ANS Panel as reliable was not zero as stated in your letter. Instead, we found a similar proportion of studies in which an adverse effect by aspartame was described (typically at a dose above the NOAEL) and that were considered by the ANS Panel as unreliable as compared to reliable (43% vs 57%). Moreover, the relative proportion of studies produced or funded by industry and found reliable to be used in the safety assessment of aspartame to be very similar to the proportion of studies carried out using no-commercial funds, irrespective of the outcome of the study (54% vs 46%). Those findings do not support your claim that EFSA took a pro-industry views. I remain confident after reviewing our internal analysis that the analysis of the studies and the literature reported in the aspartame opinion was conducted in a scientifically rigorous manner and that there was no evidence of bias.” [65].

In other words, EFSA argued that it had been symmetrically sceptical, with respect to both putative false positives and putative false negatives. This paper is in part a response to that claim, but it is also intended to deepen understandings how some regulatory scientific panels discharge their duties.

### Section 2

#### Methods and approach

It is against the background of this contested saga that we report the results of our characterisation of the ways in which the EFSA ANS panel interpreted the individual studies that were included in Section 3.2 of the ANS Panel’s December 2013 review; the section is headed ‘Toxicological data of aspartame’; it extended from pages 56 to 102. All the studies and documents are listed in full in EFSA Panel report (available at https://efsa.onlinelibrary.wiley.com/doi/epdf/10.2903/j.efsa.2013.3496, see pages 151–170).

The goal of this investigation was to establish if the ANS panel even-handedly tried to identify possible unreliable positives and unreliable negatives, or whether it asymmetrically focused more on one than the other? That issue is important because an asymmetry would constitute evidence of bias. If greater effort had been devoted to identifying and discounting false negatives that would indicate bias against aspartame, and in favour of consumer protection, whereas if greater effort had been devoted to identifying and discounting false positives then the bias would have favoured commercial interests to the detriment of consumer protection. Symmetry would imply that the ANS panel was neutral as between those two competing interests. It is important however to acknowledge that a symmetrical perspective need not entail equal numbers of true and/or false positives and true and/or false negatives. That would depend, amongst other things, on the truth regarding the safety or otherwise of aspartame.

A toxicological risk assessment requires more than the collection of data. The data generated by empirical studies need to be evaluated and interpreted. All studies have shortcomings, but some are significantly more robust and reliable than others. That is especially the case when the data derive from studies of model systems, such as laboratory rodents or microbes in glass dishes rather than from clinical or epidemiological studies of people. The relevance of, for example, the results of a rodent feeding study to the probable effects on people can never be taken for granted. Not all studies are equally relevant, and judgements of extrapolative relevance are firstly unavoidable and secondly never determined solely by reference to the results of the study under consideration.

Judging extrapolative relevance is closely related to judgements about the existence, extent and implications of uncertainties, and about how the benefit of the consequent doubts should be allocated. This is the basis for the contrast between so-called ‘positive’ and ‘negative’ regulatory list systems. With a negative list systems chemicals are assumed to be safe until shown to be harmful, while a positive list system assumes that they may be risky unless and until sufficient evidence of safety is provided. The EU is supposed to have a positive list system for many categories of food additives, including intense sweeteners. With respect to Section 3.2 of the ANS panels’ December 2013 review of the safety of aspartame, three main types of answers might be forthcoming to the question of whether the ANS panel's treatment was biased.

1. If the ANS panel’s treatment was symmetrically concerned with, and sensitive to, identifying apparently ‘positive’ findings of toxicity and ‘negative’ findings then its perspective could be characterised as neutral as between commercial and consumers’ interests. For the purposes of this appraisal, symmetry was the null hypothesis.
2. If the panel was more concerned to detect and discount putative unreliable negatives than unreliable positives then it could be characterised as favouring the interests of consumers over commercial ones.
3. If, however, the panel was more concerned to detect and discount putative unreliable positives than unreliable negatives then it should be characterised as favouring commercial interests over those of consumers.

We have used, as the indicators of symmetry or asymmetry, firstly the quantitative frequency with which studies were variously deemed by the ANS panel to be reliable or unreliable, and secondly the qualitative stringency of the hurdles that had to be satisfied for the panel to deem a putative positive as a reliable positive or an apparently negative study as a reliable negative. The features and severity of those hurdles were sometimes explicit in the panel’s text, but often they had to be inferred from the comments in the text, some of which were rather enigmatic, and from the subsequent discussion leading to the Panel’s conclusions.

The data for this analysis were obtained solely by focussing on the studies cited and discussed in Section 3.2 of the December 2013 report (pages 56 to 101), which was entitled ‘Toxicological data of aspartame’. This analysis therefore does not cover parts of the document, such as Section 2.8, that reviewed evidence for an ‘exposure assessment’ or Section 3.1 on ‘Absorption, distribution, metabolism and excretion of aspartame’. The corresponding discussions of the toxicity of aspartame-derived methanol or diketopiperazine (DKP), in Section 7 pp. 127–133, are also outside the scope of this analysis. While our analysis is not exhaustive, it focusses on a pivotal section and is sufficient to sustain robust and relevant conclusions. There is also no reason to think that Section 3.2 might be unrepresentative of the document as a whole; though if it were, that would be no less problematic. A detailed tabulation of all of the studies cited by the ANS panel in Section 3.2 of its December 2013 report is provided in Additional file 2.

Those studies are allocated to the 7 categories, which are listed below in Table 1, in Additional file 1. The resulting statistics are provided in Tables 2 and 3 below.

**Table 1:** analytical categories used in this report

		Category
1	rP	Study result(s) deemed reliable by the panel as indicating adverse effects on humans, ie reliably positive
2	uP	Study result(s) deemed unreliable by the panel as indicating adverse effects on humans, ie unreliable positive
3	rN	Study result(s) deemed reliable by the panel as indicating no adverse effects on humans, ie reliable negative
4	uN	Study result(s) deemed unreliable by the panel as indicating no adverse effects on humans ie unreliable negative
5	Cont	Contradictory (when comparing the wording in the main report with text in the Appendices of the ANS panel's report)
6	ELlow	Study indicating NOAEL at/or below 4000 mg/kg bw/day
7	E Lhigh	Study indicating adverse effects, but only at doses above 4000 mg/kg bw/day

**Table 2:** ANS panel’s interpretation of the reliability of studies for those that had, and had not, indicated possible harm, by number of studies

	Number of studies reviewed	Number treated as reliable	Number treated as unreliable	Number of studies on which the panel was self-contradictory
Studies not indicating possible harm	81	62	19	2
Studies indicating possible harm	73	0	73	8

**Table 3:** Numbers in Categories 6 and 7

	Number of studies
ELlow = Studies implying a NOAEL less than 4000 mg/kg bw	16
ELhigh = Studies indicating adverse effects but only at doses greater than 4000 mg/kg bw/day	5

#### Criteria of categorisation

The following analysis has been constructed by developing two distinct binary characterisations, firstly the tenor of the authors’ conclusion of their reports of their studies and secondly whether the ANS panel had subsequently deemed those studies to be reliable or as unreliable. The first categorisation differentiates the studies into two main groups: those for which the authors provided some evidence of adverse effects on the one hand and those providing no evidence of adverse effects on the other.

As no animal toxicology studies, of which we are aware, has shown adverse effects in all types of cells, tissues or systems, our default assumption has been to categorise studies as ‘positive’, ie indicating possible harm, if the evidence concerned one or more parameters. On the other hand, studies have been categorised as negative if no indications of harm were reported by the authors. The panel’s approach was to allow one important exception to that interpretative criterion. The panel would sometimes report evidence of adverse effects in particular studies that only occurred in animals that had received high doses of the test material [66]. The panel chose to interpret those studies as providing quantitative indicators of lower levels of exposure at which such adverse effects were not apparent; it referred to them in toxicologically-conventional terms as indicating ‘no observed adverse effect levels’ (or NOAELs), which are reported in terms of the dose measured in milligrams per kilogram of the body weight of the recipients per day (or mg/kg bw/day), The panel followed orthodox official practices by interpreting such levels as if they were thresholds of exposure below which adverse effects did not occur, in that variety of that species, or (at any rate) a level at which adverse effects were not observed.

Food additive policies typically invoke what are called ADIs (or acceptable daily intakes), where ADIs are defined as the lowest observed NOAEL, in the most sensitive laboratory species, divided by a ‘safety factor’ (or SF). The most commonly quoted safety factor is ‘100’, ostensibly on the assumption that a factor of 10 accommodates the difference between average humans and average laboratory animals, and another factor of 10 accommodates the variations amongst humans [67]. The prevailing ADI for aspartame in the EU is 40 mg/kg bw/day, as the ANS panel applied a safety factor of 100. We therefore took particular note of which studies provided evidence of adverse effects below or above the designated NOAEL of 4000 mg/kg bw/day. It is important to appreciate, however, that most professional toxicologists think that it is inappropriate to set an ADI for compounds deemed to be carcinogenic by reference to an estimated NOAEL from an animals study. This is because one mechanism by which compounds can act as carcinogens is by damaging the DNA in the nuclei of cells, that is by being genotoxic, and for genotoxic carcinogens one molecule could be sufficient to initiate or promote tumours, and therefore no level of exposure should be deemed acceptably safe. Given that three rodent studies conducted by the Ramazzini institute had provided evidence indicating that aspartame is a rodent carcinogen, the decision of the ANS panel to allocate an NOAEL to aspartame and then to ascribe an ADI to it was problematic.

We gave serious consideration to the possibility of categorising studies only showing evidence of harm at doses above 4000 mg/kg bw/day as providing positive evidence of harm, if only at the higher doses, but for the purposes of this analysis we chose to categorise such studies in line with the ANS panel’s portrayal of them, as not showing adverse effects at levels below the NOAEL. For completeness, our analysis nonetheless includes an estimate of the number of studies in which adverse effects were only evident at levels of exposure above 4000 mg/kg bw/day.

In the following discussion, individual studies and/or papers are referred to either by reference to the family name of the first author, along with the year of publication, or in terms of the number assigned to the study by G D Searle in its submission to the US FDA in the 1970s. All of the early Searle studies were assigned numbers prefaced by the letter E, and that is how the ANS panel referred to them. This paper follows that practice.

To differentiate studies in terms of whether or not they indicated possible harm, we relied in the first place on the ANS panel’s account. For example, when the panel said of studies E97 and E101 that: “Aspartame was tested for mutagenicity in [5] *Salmonella typhimurium* strains … Aspartame was not mutagenic in this test system, either in the absence or in the presence of the metabolic activation system” (p 59) it was straightforward to categorise those studies as not indicating harm. On the other hand, when the panel said of Halldorsen et al. 2010 (“A large prospective cohort study … based on data from the Danish National Birth Cohort [that] investigated associations between consumption of artificially sweetened and sugar- sweetened soft drinks during pregnancy and subsequent pre-term delivery” (p 86)) “Statistically significant trends were found in the risk of pre-term delivery with increasing consumption of artificially sweetened drinks (both carbonated and non-carbonated), but not for sugar-sweetened drinks. In the highest exposure groups (≥ 4 servings/day) the odds ratios relative to non-consumption were 1.78 … and 1.29 … respectively for carbonated and non-carbonated artificially sweetened drinks.” (p 87) it was straightforward to categorise that study as providing some indication of possible harm from artificially sweetened beverages, a market that aspartame currently dominates.

Categorisation was not, however, always straightforward because in some cases the panel’s text was unclear, evasive or even self-contradictory. In all of those cases we checked the text of the original publication, to identify any toxicological effects that were reported by the researchers. In a few cases we made our own interpretative judgements about whether or not the findings reported by the author(s) should be characterised as adverse. We did not check all the original documents because, for the purposes of this analysis, whenever the panel reported prima facie evidence of adverse effects that was sufficient reason to categorise the studies accordingly.

To differentiate studies, in terms of whether or not the panel deemed them reliable or unreliable, we drew in the first place on the panel’s text. For example, when the panel said of study E81 that it: “… noted some discrepancies in description of doses … and that the test system employed has not received further validation and is presently considered obsolete and therefore, the results of the study were not included in the assessment…” it was straightforward to categorise the panel’s judgement; the panel deemed it unreliable.

Similarly, when the panel commented of E43 that: “The methods implemented were thought to be sufficiently robust to support the results reported” (p 209) it was straightforward to categorise the study as one that the panel had deemed reliable. On other occasions, however, the panel failed to provide any explicit indication as to whether or not particular studies were deemed reliable. In such cases (eg E55, 1973, 3.2.5.1.2 page 75) categorisations were based on an exercise of informed judgement. For example, where the panel suggested in passing that some symptoms emerged that might possibly have been related to exposure to the test compound, but subsequently never again mentioned that evidence, particularly not in the summary and conclusions of the relevant section, we judged that the putatively positive evidence had been discounted, and deemed to be unreliable.

The seven categories into which we differentiated the studies, and the panel’s interpretations of their results, are set out in Table 1.

The reasons for selecting the first four of those categories should be self-evident; the others deserve brief explanations. Categories 5, 6 and 7 were not ones that we had expected would be required, but the need for them emerged as the panel’s account of each individual study was examined. Category 7, ‘Cont’ was necessary because in several cases the main texts in Section 3.2 were subsequently contradicted by comments included in one of the appendices.

Our quantitative analysis, set out in Table 2, did not include every single study or paper referred to in Section 3.2 of the panel’s December 2013 report. For example, to minimise avoidable double counting we omitted Iwata 2006 because it was not a separate study, but a re-evaluation of the previous Ishii et al. 1981 study. It is also important to note that our categorisations were not always exclusive. For example, we assigned E81 and E44 to both uN and Cont, because the text in the appendix contradicted the wording of the main text. In adopting that categorisation, the wording in Section 3.2 is treated as representing the panels’ definitive judgement while the comment in the appendix is treated as a secondary qualifier. E11, E9, E39, E54, E55, E56, E90 and McAnulty et al. 1989 belong in both uP and ELlow. E47, E63, E51, E52 and E79 belong in uP, Cont and ELlow. E48 belongs in uP and Cont. Reynolds et al. 1976 belongs in uP and uN. Bunin et al. 2005 belongs in uP and ELhigh. Consequently the aggregate count of the number of exemplifications of the 7 categories in Table 2 is greater than the total number of individual studies.

## Results

Using those criteria, in relation to Section 3.2 of the December 2013 report of the ANS panel, we generated the descriptive statistics shown in Tables 2 and 3. Table 2 specifies the numbers of studies that provided either no evidence that aspartame is harmful (in the first row, for which we identified 81 studies) or evidence of possible harm (in the second row, for which we identified 73). Those totals are partitioned into two further columns, namely those that the Panel deemed to be reliable and those it deemed unreliable; thereby providing figures for Categories 1–5.

The figures show that, while the ANS panel deemed ~ 84% of ‘negative’ studies to be reliable, it deemed every single one of the putative ‘positive’ studies as unreliable, ie the panel discounted 100% of the evidence of harm. Those figures constitute prima facie evidence of asymmetry. On the basis of its interpretation of the studies, the panel concluded that the ADI for aspartame of 40 mg/kg bw need not be changed. A tabulation of the individual studies from which those figures were derived is available as Additional file 1. Table 3 provides corresponding numbers for categories 6 and 7.

While those figures may be starkly revealing, they do not provide a comprehensive picture of the problematic features of the panel’s treatment of the putative toxicity of aspartame. Other considerations emerge from a careful scrutiny of the panel’s text, and it is to those problems that this discussion now turns. The discussion contextualises them by reference to guidance provided to all its risk assessment panels by the Board of EFSA.

### EFSA’s 2009 benchmarks of performance for all scientific risk assessment panels

The Commission’s request of EFSA to review all the evidence relating to the safety or toxicity of aspartame was issued in May 2011, and consequently the panel deliberations and conclusions ought to have conformed to some procedural guidance that had been issued by EFSA’s Scientific Committee in 2009, which referred to: ‘Transparency in the Scientific Aspects of Risk Assessments’. That guidance stipulated that:

" … scientific outputs must be transparent with regard to the data, methods of analysis and assumptions that are used in the risk assessment process …

- *Transparency is needed in all parts of the risk assessment* …
- *The sources of all data* used for the assessment, including unpublished data and personal communications, *must be referenced and … evaluated to determine their quality and relevance to the assessment*. *These should be reflected in the relative weight given to them in the assessment and taken into account in the overall evaluation of uncertainty* …
- The inclusion/exclusion criteria applied to the data should be explained and described within the risk assessment. *If data are excluded, this should be stated along with the rationale for their exclusion*." …
- *All assumptions should be documented and explained*. Where alternative assumptions could reasonably be made, the related uncertainties can be evaluated together with other uncertainties …" [68].
  (Emphases added)

In the panel’s December 2013 treatment of aspartame, all of those guidelines were breached. The following discussion substantiates those assertions, and compares the criteria of interpretation applied by the ANS panel to particular studies.

### Inclusions, exclusions and asymmetries

Although the panel did provide an account of the criteria of inclusion and exclusion that it purported to have used, the meaning of that text was vague [69], leaving the panel with considerable discretion over what to treat as evidence and what to exclude – and those discretionary exercises remained both unacknowledged and asymmetric. Evidence was often selectively excluded without due acknowledgements or explanations, thereby failing to comply with the 2009 guidance to EFSA’s scientific committees on ‘Transparency’.

One important issue concerns which reports and documents were deemed suitable for inclusion in the panel’s review and which were omitted. The discussion above highlighted the fact that detailed documentary evidence is in the public domain indicating that at least 15 of the early studies on aspartame, and of its breakdown product DKP, conducted in the laboratories of Searle, and its sub-contractor Hazleton Laboratories, were incompetently conducted and misleadingly reported, thereby concealing the incompetence. Several commentators have long argued that the defects in those studies and their reports were so serious that they should never have been accepted, and needed to be repeated and conducted properly [70].

The dossier that Millstone provided to the ANS Unit in October 2011 included the relevant documentary evidence in relation to the account provide in Section 1 above. An anomalous and problematic feature of the panel’s December 2013 report is that, while it cited passages from documents suggesting that any shortcoming in those studies were inconsequential, it failed to acknowledge, let alone engage with, the contrary evidence, such as the reasons provided by Verrett and Gross to the 1987 Congressional hearing, explaining why reassuring narratives concerning those 15 studies were profoundly flawed. The panel mentioned the report of the UAREP, referring to it “… the authentication review of selected materials submitted to the Food and Drug Administration …” [71], but it failed to cite or engage with Gross’s explanation of why the UAREP document was not a genuine ‘authentication’ or with Verrett’s contention that none of those 15 studies could be deemed reliable. Nonetheless, all 15 of those studies were included in the ANS’s analysis, and treated as if they were unproblematically reliable, although as Verrett had explained, they were ‘woefully inadequate’ and ‘worthless’. The panel’s failure to cite or discuss the evidence from eg Gross and Verrett entails that the panel’s assessment was neither thorough nor reliable.

In 1996 Olney et al. published a paper highlighting the increase in the incidence of an aggressive type of brain tumour in US adults following the introduction of aspartame [72]. There are several reasons why that report deserves to be deemed credible. Firstly, the type of central nervous system tumour found to be increasing most rapidly in the USA is the same kind of lesion as was found in one of the animal studies conducted on aspartame in the 1970s [73]. Secondly, Olney et al. drew attention to a study by Shephard et al., in which they simulated in vitro the conditions that can occur in the human digestive tract, especially the conditions that result in the nitrosation of dietary constituents. They reported that nitrosated aspartame had significant mutagenic action [74]. That is important because it suggests not only a mechanism by which aspartame could act carcinogenically, but also why the interval between the compound’s introduction and the elevation of US brain cancer rates was so brief. The paper by Olney et al. was omitted from the panel’s discussion of human epidemiology (Section 3.2.7.1.3) though it was mentioned in Section 2.7.1, where the panel cited and endorsed a 2002 report from the French food agency (AFSSA) that said: “… Olney et al. (1996) did not provide any scientific evidence of a relationship between exposure to aspartame and the appearance of brain tumours.” [75] But that remark was misleading because while it did not provide proof of such a relationship, it certainly provided evidence; which illustrates the panel’s practice of discounting any evidence of possible harm falling short of causal proof.

### Puzzling anomalies – inconsistent and unacknowledged assumptions

Table 3 above indicates that in relation to 10 studies the panel contradicted itself, when comparing the main body of the text with the appendices. The following paragraphs highlight several examples.

Study E44 [76] was a so-called ‘host-mediated mutagenicity test’ in which rats were injected with a microorganism that could reveal induced gene mutations, and then tested to see if aspartame is a mutagen. In relation to that study, which in our analysis has been categorised as not indicating harm, the panel criticised the test because: “… the test system employed has not received further validation and it is presently considered obsolete, and therefore the results of the study were not included in the assessment.” Yet in Appendix H on page 210, the text states that: “The methods implemented were thought to be sufficiently robust to support the results reported.” A similar problem arises in relation to E81 for which the text in Sec 3.2.3.1 on page 59 suggests that the results of study were not included in the assessment, while Appendix H page 210 also indicates that the methods were thought sufficiently robust. In those two cases, the panel’s comments were contradictory.

In relation to E54, which was a developmental toxicity study on rabbits, the panel reported: “… a significant decrease in fetal body weight and skeletal anomalies … [yet] … the Panel concluded from these observations that the developmental effects on body weight and skeletal development reported in the aspartame feeding studies may be caused by the significant depression of feed consumption in the high dose group.” [77] They chose to discount the indication of adverse effects in the ‘high dose’ group even though: “The actual aspartame doses were reported to be 1880 and 1870 mg/kg bw/day for the intended 2000 and 4000 mg/kg bw/day groups, respectively.” But as the intakes of the two groups were so similar, the panel’s attribution of decreased fetal body-weight to a difference in consumption was flawed. In our analysis this study was categorised as indicating possible harm, though it had in effect been deemed unreliable by the panel.

In relation to E55, which was another developmental toxicity study on rabbits, the panel failed to comment on its findings or reliability. In Appendix I page 229, however, we learn that: “Feed consumption decreased by up to 29% in the high dose group” [78]. That change may well have been a consequence of a reduction in the palatability of the diet to the rabbits in the high dose group. Nonetheless the panel problematically portrayed the highest dose of 4000 mg/kg/bw as a NOAEL. Our analysis categorised this study as not indicating possible harm, as it did so only at a high dose, but the panel failed to provide an explanation of why the reduction in food consumption was not counted as an adverse effect and therefore why the NOAEL in this study was not specified at a dose lower than 4000 mg/kg/bw.

In relation to E62, which was another developmental toxicity study on rabbits, the panel “… noted that the actual dose to which the rabbits were exposed did not exceed 1880 mg/kg bw/day (range 1160-1880 mg/kg bw/day) when the intended dose was 4000 mg/kg bw/day (administered as 4.8 % (w/w [weight for weight]) aspartame in the diet). This was a result of the considerable decrease in feed intake observed in the pregnant rabbits … in this dose group. … as such the Panel noted that the actual aspartame doses in the low and high aspartame dose groups were often comparable” [77]. In Appendix I, page 229, however the text reports that at the ‘high dose’, 3 fetuses exhibited ‘minor malformations’, yet the panel deemed 2000 mg/kg bw/day as a NOAEL, but if the doses were the same in the ‘high’ and ‘low’ dose groups, then 2000 mg/kg bw/day was a level at which adverse effects were observed, and so portraying 2000 mg/kg bw/day as a NOAEL was a misrepresentation. In our analysis this study was categorised as indicating possible harm, although it was deemed by the panel to be unreliable.

In relation to E90, a developmental toxicity study in rabbits, the panel explained that in this study: “A number of animals died spontaneously during the study … mainly due to misdosing … No abortions were detected in the control, mid dose and L-aspartic acid groups, two abortions in the low dose aspartame group and 24 abortions were observed in the high dose aspartame group (a significant increase compared to controls) and four in the L-phenylalanine group…Since the decrease in body weight started several days before (13 and 18 days) the abortions (28 days), the authors concluded that abortion was a consequence of significant and rapid body weight loss caused by decreased feed consumption” [79]. Firstly, the panel accepted the authors’ reasons for attributing the abortions to maternal body-weight loss, even though both could equally reasonably have been interpreted as adverse toxicological effects. Secondly, Appendix I (page 231) portrayed the low-dose level of 1000 mg/kg bw/day as a NOAEL, but it was problematic to portray two abortions as ‘no adverse effects’, particularly without reference to any estimate of possible statistical significance. Consequently in our analysis this study was categorised as indicating possible harm, though deemed unreliable by the panel.

In relation to Brunner et al., which was a reproductive study on rats, the panel said: “Increased offspring mortality was observed in rats fed the highest aspartame dose and in the phenylalanine-exposed animals” [80]. So while the death of infant rats deserved to be deemed as adverse, the panel claimed that it: “… agreed with Brunner et al …” implying that the adverse effects should be discounted as only occurring at highest doses. The problem is that that is not what the original paper asserted. It said: “… the addition of … aspartame (6% by weight) … to the standard diet of rats has a profound effect on early postnatal development. That the majority of adverse effects of Asp 6%…are transmitted during the period of lactation was clearly shown in a cross-fostering experiment … Conversely as 6% administered during lactation, but not earlier, resulted in high mortality and somewhat retarded development similar to that observed in the offspring of dams receiving Asp 6% continuously from prior to breeding ….” [81]. Moreover as Magnuson et al. observed: “… this difference was statistically significant.” [82]. Consequently, in our analysis this study was categorised as indicating possible harm, though it was deemed unreliable by the panel.

### Low-hurdle for the acceptability of negative studies

In section 3.2.3.1 on genotoxicity, Searle studies E97 and E101 using *salmonella* bacteria, were reported by the panel as having used methods that failed to comply with the conventional requirements for ‘good laboratory practice’, yet the December 2013 report of the ANS panel did not discount the findings from them. We, by contrast, would count them as unreliable for precisely those reasons.

In relation to E4, a sub-chronic rat feeding study, with only 5 animals per dose group, lasting only 9 weeks, no adverse effects were reported by the panel except for “… reduced body weight gain …” [83]. The panel reported: “No *significant* treatment-related changes in haematology, clinical chemistry, urinalysis were recorded and no treatment-related changes in relative organ weight or pathology were observed. Reduced body weight gain was noted for both treatments and both sexes (though less marked in females) …” [83]. (emphasis added) The panel’s use of the word ‘significant’, when combined with its failure to comment on the very small size (ie 5) of the test groups, reveals a willingness to accept a very weak negative study as if reliable, despite its obvious weaknesses. Other examples of where the panel treated negative studies as if they were reliable, despite the small size of the experimental samples, are provided for example by E86 (Sec 3.2.4.1 p 69, which examined brain tissue cells) which was treated as a reliable negative [84], despite having only 5 dogs per dose group, and Sasaki et al. 2002, which was treated as a reliable negative despite using only 4 mice per group, and which received only a single dose [85].

In relation to E51, the panel suggests that no embryotoxic effects were observed in a reproductive study on rabbits. The text also explained that: “The study was confounded by poor health of the animals and the gavage technique issues. As a result, maternal mortality was high. The administration of aspartame was associated with depression of feed consumption by up to 40%.” [86]. In Appendix I, however, the text reveals that the number of abortions and maternal deaths in the high dose group were double those in the control group [87]. That information contradicts the claim that no embryotoxic effects were observed. Furthermore the dose at which such effects emerged was 2000 mg/kg bw/day, so the suggestion that that figure could be deemed a NOAEL is problematic. Yet again negative indications were treated as reliable, while positive findings were discounted as unreliable.

In relation to E43, which was a study in rats investigating chromosome aberrations in bone marrow cells, the panel complained that ‘no mitotic index’ had been determined, and therefore deemed the study “limited” [88]. A mitotic index is an estimate or measure of the number of cells per unit (usually 1000 cells) undergoing cell division in a specific time period; it provides an estimate of the rate of tissue growth. In Appendix H the panel commented that: “The mitotic index should be determined as a measure of cytotoxicity in at least 1000 cells per animal for all treated animals (including positive controls) and untreated negative control animals. **Overall judgement**: The methods implemented were thought to be sufficiently robust to support the results reported” [89]. (emphasis in the original) In other words, even though the mitotic index had not been estimated, the study had been deemed ‘sufficiently robust’. By contrast, Mukhopadhyay et al’s findings, in a study of chromosomal aberrations in mice, using a blend of aspartame and acesulfame K, and which indicated adverse effects, was discounted because no mitotic index had been determined and because a mixture of sweeteners was used [90]. But if the lack of a mitotic index did not count against E43 why did it count in this case? The panel’s failure to acknowledge that blends of aspartame and acesulfame K are often used in food and beverage products (eg Coke Zero) was also conspicuously absent [91]. The panel therefore invoked a ‘mitotic index’ criterion inconsistently. Representatives of the sweetener industry have also claimed that while a blend of aspartame and acesulfame K is significantly sweetener than the sum of their separate sweetness, ie they act synergistically on our taste buds, they are entirely confident that toxicologically they cannot act synergistically [92].

When responding to seemingly positive evidence of toxicity the ANS panel never set a requirement, or issued requests, for follow-up studies, or further information. It used the lack of follow-up confirmation as grounds for discounting evidence of possible harm, but not as grounds for requiring further tests; a precaution that had at least sometimes been taken previously by the UK, European and WHO advisory panels.

### (Unreachably) high hurdles for ‘positive’ studies indicating adverse effects – assumptions not acknowledged but revealed

Official toxicologists routinely use so-called ‘no observed adverse effects levels’ (or NOAELs) in animal studies as a basis for setting an ‘acceptable daily intake’ (or ADI) for regulated compounds, simply by applying a ‘safety factor’ which is often 100. If the protection of public health is the primary goal of policy, the selected NOAEL should be no higher than the dose below which no adverse effects were observed in the most sensitive parameters of the most sensitive variety of the most sensitive laboratory species tested.

For reasons that have not been explained, the ANS panel discounted several pieces of evidence indicating that the prevailing NOAEL for aspartame of 4000 mg/kg bw/day was too high. Section 3.2 included at least 12 examples of the panel noting, but then discounting, evidence of adverse effects, and therefore NOAELs, below 4000 mg/kg bw/day. For example, E39 [93], which studied the effect of aspartame on peri and postnatal development in female rats, was reported as indicating a NOAEL of 2000 mg/kg bw (as did E9, E47, E48, E53, E54 and E62) while the lowest reported figure was 750 mg/kg bw in study E79 [94]. For E62 the panel even generously accepted 2000 mg/kg bw as a NOAEL, despite reporting ‘minor malformations’ in infant rabbits at that dose [78]. Those examples illustrate the forgiving generosity the panel showed towards studies and dose levels at which ostensibly adverse effects were observed and reported. Those examples thereby reveal the demanding heights of the hurdles that studies providing evidence of possible harm would have had to have reached before the panel might have deemed them relevant and reliable.

Another example of the panel discounting evidence of possible harm by implicitly setting a very high hurdle is provided by two studies of the consequences of nitrosating aspartame, namely Meier et al. 1990 and Shephard et al. 1993. The panel discounted the evidence from those studies by suggesting that the conditions for nitrosation were ‘harsh’, without acknowledging that nitrosation is precisely the kind of process that happens during human digestion, and that conditions in the human stomach, especially after consuming highly acidic cola beverages, are similarly ‘harsh’ [95].

In relation to Searle study E20, a 2-month oral administration study in 10 male and 10 female rats, the panel discounted “… a significantly higher liver to body weight ratio … for the high dose group males compared to controls” [96]. But the ‘high dose’ level was only 125 mg/kg bw/day [97]. Changes were discounted as being neither systemic nor pharmacological, which was implicitly invoking a very high hurdle.

In relation to Searle study E87, which examined rat brain tissue from E33–34 and E70 looking for intracranial neoplasms, the panel reported that tumours were found, which were random with respect to dose and gender; the panel discounted the tumours as unrelated to aspartame [98]. What the panel failed to acknowledge or explain was why it (implicitly) assumed that cancers in rats only count if they affect males and females to a similar extent, and only if the dose-response relationship is monotonic. Both those assumptions implicitly favoured commercial over public health interests, and are scientifically contested by reference to both empirical evidence and theoretical understandings. The panel’s treatment of E87 therefore failed to conform to the injunction to EFSA panels that: “All assumptions should be documented and explained …” [99]. Moreover it reveals the asymmetry of the height of the implicit hurdles, and therefore a failure to be even-handed.

In relation to Searle study E20, an 8-week rat feeding study using 50 animals of which 10 served as controls, the panel reports that notwithstanding “… organ weights were unaltered except in the high dose males where a significantly higher liver to body weight ratio was observed for the high dose group males compared to controls” [100]. In Appendix G the text indicated: “… increased terminal blood sugar at 125 mg/kg bw/day but no other significant changes in clinical chemistry …” as well as “… no unequivocal effect on bodyweight or food consumption.” [101]. In other words, the liver-to-body-weight ratio was significantly disturbed in the high dose males, which in such a small group (5 animals) suggests a systemic problem, which nonetheless the panel discounted. Characterising and discounting effects on body weight and food consumption as not ‘unequivocal’, and implicitly demanding unequivocal findings from a study with a group size of 5, provides further examples of the panel raising the bar for positive evidence, despite keeping it very low for reassuring evidence and studies.

When the panel discussed Ishii et al’s 1981 2-year chronic toxicity and carcinogenicity rat study [102]. it reported ‘dose related changes’ including ‘focal mineralisation of the renal pelvis’, but those findings were discounted as of “… minimal toxicological significance …” . But several other small studies that failed to find tumours were portrayed as if toxicologically significant and reliable, revealing a further example of asymmetric benchmarks of appraisal and criteria of interpretation. Indeed the panel: “… noted that the study provided information on the lack of toxicity of aspartame when administered in conjunction with DKP” [102]. This is an example of the panel’s willingness to portray the absence of evidence as if it constituted evidence of absence, which is always an invalid inference. The panel also failed to acknowledge any of its underlying assumptions, thereby failing to follow the procedural stipulations that the EFSA Board had specified.

In relation to Halldorsson et al. 2010, a prospective cohort study on the association between intakes of artificially sweetened soft drinks and pre-term delivery, the panel reported that “Statistically significant trends were found in the risk of pre-term delivery with increasing consumption of artificially sweetened drinks (both carbonated and non-carbonated), but not for sugar-sweetened drinks.” [103]. Nonetheless, the panel declined to treat those findings as reliable, noting that: “… the prospective design and large size of the study sample were major strengths, and there were no important flaws in the methods used. However, risk estimates may have been inflated by residual confounding (including by year of delivery). No account was taken of other dietary sources of methanol, and use of aspartame specifically was not distinguished from that of other artificial sweeteners). Therefore, given these limitations, the Panel agreed with the authors who concluded that replication of their findings in another setting was warranted.” [104]. The panel’s remark that the results: “… may have been inflated by residual confounding …” [104] was shallow and opportunistic. The results might have been affected by unidentified confounding factors, but they may not have been, and the fact that residual confounding was a logical possibility cannot constitute sufficient grounds for disregarding the indications of harm, especially as the panel never raised the possibility of over-adjusting for confounding variables in the numerous negative studies that were treated as if reliable.

The next study the panel discussed was Englund-Ögge et al. 2012, which was portrayed as if it had been an attempt to replicate the study by Halldorson et al. [105]. The panel said that: “No significant trends were found in the risk of pre-term delivery with increasing consumption either of artificially sweetened drinks or of sugar-sweetened drinks. [but] Small elevations of risk were observed with higher consumption of artificially sweetened soft drinks, but after adjustment for covariates, these reached statistical significance only when categories of consumption were aggregated to four levels, and then the odds ratio for the highest category (≥ 1 serving/day) was only 1.11 (95 % CI 1.00-1.24) in comparison with non-consumption.” [106]. In other words ‘after adjustment for covariates’ the Endlund-Ögge et al. study reported similar findings, but only more weakly than Halldorson et al.

In relation to those two studies the panel remarked that: “Both Halldorsson et al … and Englund-Ögge et al … appear to have been well designed and conducted. Noting this, the Panel concluded that even at high levels of exposure to artificially sweetened soft drinks the risk of pre-term delivery is likely to be small, if any. The observed associations could be a consequence of uncontrolled residual confounding, and the inconsistencies in the patterns of association reinforce this uncertainty.” [106]. The panel interpreted the results of Englund-Ögge et al. as if they refuted those of Halldorsson et al., when it would have been no less reasonable to interpret Englund-Ögge as supporting Halldorson, though with a weaker signal, which constitutes another example the panel’s underlying unacknowledged asymmetric assumptions.

In relation to Camfield et al. 1992, which was a double-blind study with eight girls and two boys to ascertain aspartame’s relevance to their neurological seizures, the panel: “… noted that the combination of the two parameters (number and length of spike-wave bursts) into a single measure was not adequately explained, and lack of control of food and drink intake before and after dosing may have affected the results. The Panel further noted that aspartame was given in a single dose at the ADI.” [107]. But since both the frequency and severity of seizures are toxicologically significant adverse effects, integrating them into a single indicator can be revealing rather than misleading. It is also noteworthy that the panel never questioned the choice of indicators used in negative studies. Once again the panel picked on tiny imperfections in a putative positive, while in relation to negative studies appearing indifferent, not just to similarly tiny imperfections, but to substantially more serious ones.

When discussing Kulczycki’s 1986 report of a case of aspartame-induced urticaria, the panel said that it discounted all self-reported allergic-like reactions on the grounds that the European Commission’s Scientific Committee for Food had previously discounted them in 2002 as they had not been confirmed double blind [108]. Nonetheless, lower down the same page the panel acknowledged that: “Kulczycki … reported a case of aspartame induced urticaria *confirmed by double blind challenge* …” [109]. (Emphasis added) One page later the panel said: “However, the Panel cannot exclude the possibility that in rare instances individuals could be susceptible to allergic reactions following aspartame ingestion” [110]. Yet the panel treated that evidence and the possibility as if it had no implications whatsoever for the acceptable daily intake of aspartame; which leaves unanswered the question ‘acceptable to whom’: to NutraSweet, to average healthy adults, or to all likely consumers? Unfortunately no answer is provided to that implied question; instead ADIs are treated as if they were natural constants rather than value-based social judgements about how much risk should be tolerated, and by whom.

In relation to Veien et al. 2012, which was entitled ‘Systemic allergic dermatitis presumably caused by formaldehyde derived from aspartame’, the panel acknowledged that a: “… few cases of presumed systemic allergic dermatitis in patients with contact sensitivity to formaldehyde, apparently caused by the intake of aspartame in artificial sweeteners, have been described. The four patients described in the literature all had eyelid dermatitis” [111]. Those findings were then discounted by the panel on the grounds that: “… the studies available were performed on a limited number of participants.” A study with a small sample size is unavoidably insensitive and yet effects were still noted and should not have been discounted because the sample size was small.

In relation to Roberts 2001, the panel mentioned: “… case reports … published in peer reviewed journals and reports compiled by Dr H.J. Roberts and published under the title *Aspartame Disease – An ignored Epidemic* … The total number of symptoms reported from all sources was 4281, as most cases reported more than one symptom. Headache was the most frequently reported adverse effect (28.5 %), followed by dizziness and giddiness (19.2 %). Although the results of a questionnaire-based study (Lipton et al., 1989) and two double-blind out-patient investigations (Koehler and Glaros, 1988; Van den Eeden et al., 1994) employing daily doses of up to 30 mg/kg bw/day indicated a potential association between aspartame intakes and headache, it is still not possible to deduce causality, as the effect of diet has not been adequately controlled for and the interpretation of the data was complicated by a high dropout rate and a limited experimental design. The Panel noted that the number of cases is low when compared with the widespread use and that the effects were mild to moderate” [112]. (emphases added) Using the phrase ‘it is still not possible to deduce causality’ reveals that when faced with apparently positive evidence of harm from two double-blind investigations the panel invoked the astonishingly demanding hurdle of deductive proof of a causal relationship; a criterion that was radically more demanding than any or all of those utilized to interpret negative studies, for which mere plausibility was often treated as sufficient.

### Selecting 4000 mg/kg bw/day as a NOAEL for aspartame

Our comprehensive tabulation (see Additional file 2 and summarised below in Additional file 1, which in turn is summarised in Tables 2 and 3 above) identifies 16 studies that showed adverse effects at dose levels of less than, or equal to, 4000 mg/kg bw/day. This is important because, supposedly, the dose level at which a NOAEL should be specified ought to be the highest level producing no adverse effects in the most sensitive variety of the most sensitive species. Since the set of studies the panel reviewed in Section 3.2 included 12 that did show evidence of adverse effects at or below 4000, it is clear that the panel’s designation of that dose level as a NOAEL was flawed and unjustifiable. The studies that provided evidence of adverse effects at or below 4000 mg/kg bw/day are: E20, Ishii et al. 1981, E11, E9, E39, E47, E53, E54, E55, E63, E51, E52, E79, E90, McAnulty et al. 1989 and NTP-CERHR Report 2003.

### Summary of results of qualitative analysis

If all of the implicit benchmarks, which the ANS panel invoked, as grounds for discounting putative positive evidence of adverse effects from aspartame, are aggregated together they collectively imply that, for the ANS panel, nothing could count as a reliable positive unless:

1. the magnitude of the evidential difference between test and control groups satisfied the customary criterion of statistical significance, namely that there was less than one chance in 20 of it having been a random fluctuation (or a *p*-value < 0.05) (cf E2 1972; E3 1972; E44 1972; E75 1974);
2. the results were entirely unequivocal (cf NTP 2005; E9, 1972; E11, 1971; Collison et al. 2012b);
3. the findings were consistent eg consistently and monotonically dose-related (cf E21);
4. were obtained from a long-term study conducted with a large sample of people or large groups of laboratory animals (cf Abhilash et al. 2011); and.
5. entirely free of any imperfections (cf Kamath et al. 2010; Sasaki et al. 2002).

Those are some of the assumptions that would need to be adopted in any attempt to reproduce the ANS panel’s conclusions. Without those particular assumptions, the panel’s conclusions could not have been reached.

On the other hand, studies were treated by the panel as if they were reliable negatives despite numerous imperfections (eg E43) of the sort that the panel highlights when discounting putative positives. For example: “…the findings reported might be ascribed specifically to the conditions of the study…” [113].

## Discussion

This paper has provided numerous pieces of evidence showing that the implicit criteria by reference to which the ANS panel evaluated toxicological studies on aspartame were not even-handed. On the contrary, they were markedly asymmetric as between apparently positive and negative studies. In quantitative terms, the panel treated ~ 76% of apparently negative studies as if they were reliable, despite their numerous shortcomings, yet it deemed 100% of positive studies as unreliable, despite the fact that their shortcomings were often fewer and less serious than those that characterised the negative studies. This asymmetry favoured commercial and industrial interests over the protection of consumers.

The qualitative analysis showed that, while very demanding criteria were used to judge the reliability of putative positive studies, far more lax and forgiving criteria were applied to judge the reliability of apparently negative studies. If the benchmarks that the ANS panel used to evaluate the results of ‘negative’ studies had been consistently used to evaluate the results of ‘positive’ studies then the panel would have been obliged to conclude that there was sufficient evidence to indicate that aspartame is not acceptably safe. Correspondingly, if the benchmarks that the ANS panel used to evaluate the results of positive studies had been consistently used to evaluate the results of negative studies then the panel would have been obliged to conclude that there was insufficient evidence to show that aspartame is acceptably safe. In the event, it did the opposite.

The ANS’s December 2013 treatment of evidence concerning aspartame toxicity failed to conform to basic standards of scientific rigour and more specifically failed to comply with EFSA’s *Guidance of the Scientific Committee on Transparency in the Scientific Aspects of Risk Assessments carried out by EFSA*, in all of the following respects. In the following list, key words and phrases used in the EFSA Guidance are italicised.

- The output of the ANS panel’s review of aspartame was not *transparent with regard to the data, methods of analysis and assumptions* that were used in the risk assessment process.
- *The sources of all data* *used for the assessment, including unpublished data and personal communications*, were not properly *evaluated to determine their quality and relevance*. The *relative weight given to* the available evidence *in the overall evaluation* was not even-handed but asymmetric as between ‘positive’ and ‘negative’ studies. The uncertainties that were acknowledged by the panel related far more frequently to ‘positive’ studies than to ‘negative’ ones.
- The criteria of *inclusion* and *exclusion* of evidence were applied inconsistently and were neither properly *described* nor *explained.*
- When studies and *data* were *excluded* from the risk assessment the reasons were often not given, and when some were stated, they were not applied consistently.
- Numerous *assumptions* were neither *documented* nor *explained.*
- *Where alternative assumptions could* have *reasonably been made*, the panel failed to identify them, or to appraise *uncertainties* in an even-handed manner.

The assumptions that the panel implicitly made were applied in a consistently inconsistent manner, in ways that favoured commercial interests over consumer protection.

There is little point requiring animal tests and, as the euphemism has it, ‘sacrificing’ the animals unless those studies are conducted and interpreted so as to protect public health.

## Conclusion

This paper provides compelling quantitative and qualitative evidence of asymmetric criteria of interpretation and appraisal on the part of the ANS panel, but it does not provide an explanation of why such an asymmetrical approach was taken. A hypothesis that some members of the ANS panel may have had commercial conflicts of interest has been advanced [114], but as the panel’s meetings all took place behind closed doors, such hypotheses are difficult to test. An alternative hypothesis that regulatory institutions exhibit forms of institutional inertia, meaning that they are singularly unwilling to criticise their previous judgements or the judgements of other official bodies including their predecessors, is plausible but it will also remain hard to test until risk assessment processes become substantially more transparent.

On the issue of the transparency of EFSA risk assessment the chief executive of EFSA, Bernhard Url, was reported, by the commercial food industry news service NutraIngredients.com, as having said in October 2015 that:

“In a scientific process – which is what we call organised scepticism – the scientist must have the freedom to ask stupid questions that are out of the box, to challenge their peers, trial and error. … And there I’m not convinced we help the process of finding the nearest approximation of truth by putting every single question for the whole life of a scientist on Youtube … .I think that science needs parts of the process in a closed room and then many steps of the process in an open atmosphere.” But I think it also needs a protected room where they can speak completely freely, openly and challenge each other. That’s also science." [115].

Those remarks were difficult to reconcile with EFSA’s commitment to transparency and reproducibility. No-one is proposing to put the entire lives of EFSA’s scientific advisors on YouTube, but in this context the issue relates to the transparency of specific professional activities of expert advisors when providing judgements and advice to EFSA. A good case could be made for the argument that anyone who is not willing to be fully accountable for their judgements in respect of EFSA’s food safety risk assessments should be disqualified from serving on EFSA panels. Since March 2002, expert committees that advise the UK’s Food Standards Agency have been required to hold their meeting in publicly open sessions, and there is no obvious reason why the same requirement could not be applied by EFSA [116]. It would contribute substantially to enhancing EFSA’s credibility with citizens of the EU.

Url argued (see above) that EFSA’s scientific advisors must be free to ask stupid questions. What counts as a ‘stupid’ question may often be an issue about which people can legitimately disagree, but it is vitally important to ensure that members of EFSA panels are consistently asking wise and thoughtful questions that challenge the claims put before them. The discussion above suggests that the ANS panel questions were not always consistently wise or sceptical. Moreover the lack of transparency risks the continuation of bad practices.

Given the shortcomings of EFSA’s risk assessment of aspartame, and the shortcomings of previous official toxicological risk assessments of aspartame, it would be premature to conclude that it is acceptably safe. They also imply that the manner in which EFSA panels operate needs to be scrutinised and reformed.

EFSA, the European Commission, the European Parliament as well as EU consumers, have been poorly served by the ANS panel. The ANS panel may have wished to portray the December 2013 document as its ‘final report’, but that may well have been over-optimistic.

A fresh review of all the data from all the studies, as well as all other relevant scientific and policy documents, should be conducted. None of the members of that review panel should have any relevant conflicts of interest. Its conduct should be fully transparent and demonstrably compliant with all EFSA Guidance documents, and it should also be guided by bias-detection tools such as those provided by SYRCLE [117]. and CAMARADES [6], the selection and application of which should be explained and justified.

## Additional file

Additional file 1: List of studies allocated to categories 1 to 7. (PDF 462 kb)

Additional file 2: Comprehensive tabulation of all studied cited by EFSA’s ANS panel in Section 3.2 ‘Toxicological data of aspartame. (PDF 655 kb)

## Data Availability

All relevant data and material will be available on publication. Additional file 2 and Millstone’s dossier is available at http://www.sussex.ac.uk/spru/research/projects/fcs.

## Abbreviations

ADI: Acceptable daily intake
ANS Panel: EFSA’s Panel on Food Additives and Nutrient Sources added to Food
APM: Aspartame
Cont: Contradictory (when comparing the wording in the main report with text in the Appendices of the ANS report)
CoT: Committee on Toxicology, of the UK
DKP: Diketopiperazine, a breakdown product of aspartame
EFSA: European Food Safety Authority
ELhigh: Study indicating NOAEL at/or below 4000 mg/kg bw/day
ELlow: Study indicating adverse effects, but only at doses above 4000 mg/kg bw/day
EU: European Union
FAO: UN Food and Agriculture Organisation
FDA: US Food & Drug Administration
JECFA: Joint Expert Committee on Food Additives of the WHO and FAO
mgs/kg bw/day: milligrams of aspartame per kilogramme of the body weight
NOAEL: No-observed-adverse effect-level
PBOI: Public Board of Inquiry
rN: Study result(s) deemed reliable by the panel as indicating no adverse effects on humans, ie reliably negative
rP: Study result(s) deemed reliable by the panel as indicating adverse effects on humans, ie reliably positive
SCF: Scientific Committee on Food, of the European Commission
SF: Safety factor
UAREP: US Universities Association for Research and Evaluation in Pathology
UK: United Kingdom
uN: Study result(s) deemed unreliable by the panel as indicating no adverse effects on humans, ie unreliable negative
uP: Study result(s) deemed unreliable by the panel as indicating adverse effects on humans, ie unreliable positive
w/w: weight for weight
WHO: World Health Organisation
