# Supplementary material for: EFSA’s toxicological assessment of aspartame: was it even-handedly trying to identify possible unreliable positives and unreliable negatives?
Source: Arch Public Health. 2019 Jul 15;77:34. doi: 10.1186/s13690-019-0355-z (PMC6628497; doi:10.1186/s13690-019-0355-z)
Supplement: Supplementary file 1 — List of studies allocated to categories 1 to 7. (PDF 462 kb) [file 13690_2019_355_MOESM1_ESM.pdf]

Appendix 1: list of studies allocated to categories 1 to 7

Category	Study Id	Count
**1 = rP: Study result(s) deemed reliable by the panel as indicating adverse effects on humans, ie reliably positive**		**0**
**2 = uP: Study result(s) deemed unreliable by the panel as indicating adverse effects on humans, ie unreliable positive**	E3; Abhilash et al 2011; Abhilash et al 2013; Rencuzogullari et al 2004 CA; Rencuzogullari et al 2004 MN; NTP 2005 p53-haploinsufficient; Bandyopadhyay et al 2008; Kamath et al 2010; AlSuhaibani 2010 CA; Karikas et al 1998; Meier et al 1990; Shephard et al 1993; E75; E70; Ishii et al 1981; E32; Soffritti et al 2006; Soffritti et al 2007; Soffritti et al 2010; E11; E9; E10; E39; E88; E47; E48; E49; E5; E53; E54; E55; E62; E63; E51; E52; E79; E90; Mahalik and Gautieri 1984; McAnulty et al 1989; Collison et al 2012a; Collison et al 2012b; Ranney et al 1975; E 14; Beck et al 2002; E94; Christian et al 2004; Puica et al 2008; Puica et al 2009; Tutelyan et al 1990; Vences-Mejia et al, 2006; Alleva et al 2011; Simintzi et al 2007a; Simintzi et al 2007b; Kim et al 2011; Halldorsson et al 2010; Englund-Ögge et al 2012; La Vecchia 2013; Maslova et al 2013; Hardell et al 2001; Bunin et al 2005; Andreatta et al 2008; Schernhammer et al 2012; Walton et al 1993; Camfield et al 1992; Koehler and Glaros 1988; Lipton et al 1989; Van den Eeden et al 1994; Kulczycki 1986; Butchko et al 2002 Review; Novick 1985; McCauliffe and Poitras 1991; Veien and Lomholt 2012; Robert 2001, reviewed by EFSA 2010	**73**
**3 = rN: Study result(s) deemed reliable by the panel as indicating no adverse effects on humans, ie reliable negative**	E46; E84; E85; E2; E3; E20; E21; E97; E101; E40; E41; E43; NTP 2005; Jeffrey and Williams 2000; NTP 2005 micronucleus; NTP 2005 TG.AC hemizygous; NTP 2005 CDkn2A deficient; Sasaki et al 2002; E87; E86; E89; Holder 1989; NTP-CERHR Report 2003; E104; Reynolds et al 1980; E105; Magnuson 2007; EFSA 2010 Review; SCF 2002 Review; E1; E19; Lim et al 2006; E66; E110; E23; E24; E60; E61; E95; Leon et al 1989; Porikos and Van Italie 1983; E25; E67; E109; E26; Krusei et al 1987; Wolrach et al 1984; Shaywitz et al 1994a; Roshon and Hagen 1989; Saravis et al 1990; Lapierre et al 1990; Ryan-Harshman et al 1987; Pivonka & Grunewald 1990; Stokes et al 1991; Stokes et al 1994; Spiers et al 1998; Shaywitz et al 1994b; Rowan et al 1995; Schiffman et al, 1987; Szucs et al 1986; Garriga et al 1991; Geha et al 1993	**62**
**4 = uN: Study result(s) deemed unreliable by the panel as indicating no adverse effects on humans ie an unreliable negative**	E81; E12; E44; Rencuzogullari et al 2004; Bandyopadhyay et al 2008; Durnev et al 1995; Mukhopadhyay et al 2000; AlSuhaibani 2010 SCE; Rencuzogullari et al 2004 SCE; E27; E35-36; E28; Lennon et al 1980 rats; Lennon et al 1980 hamsters; E15; Haque and Mozaffar 1993; Gallus et al 2006; Bosetti et al 2009; Cabaniols et al 2011;	**19**
**5 = Cont: Contradictory (between appendix and main body) reports**	E81; E44; E47; E48; E5; E53; E63; E51; E52; E79;	**10**
**6 = Ellow: Study indicating NOAEL at/or below 4000mg/kg bw/day**	E20; Ishii et al 1981; E11; E9; E39; E47; E53; E54; E55; E63; E51; E52; E79; E90; McAnulty et al 1989; NTP-CERHR Report 2003;	**16**
**7 = Elhigh: Study indicating adverse effects only at doses above 4000mg/kg bw/day**	E33-34; E70; Brunner et al 1979; Lennon et al 1980; E14	**5**
