# Supplementary material for: EFSA’s toxicological assessment of aspartame: was it even-handedly trying to identify possible unreliable positives and unreliable negatives?
Source: Arch Public Health. 2019 Jul 15;77:34. doi: 10.1186/s13690-019-0355-z (PMC6628497; doi:10.1186/s13690-019-0355-z)
Supplement: Supplementary file 2 — Comprehensive tabulation of all studied cited by EFSA’s ANS panel in Section 3.2 ‘Toxicological data of aspartame. (PDF 655 kb) [file 13690_2019_355_MOESM2_ESM.pdf]

Appendix 2: Comprehensive tabulation of all studied cited by EFSA's ANS panel in Section 3.2 'Toxicological data of aspartame'

Citation to source	ANS Panel's Category	Location in EFSA report, Section & page	Author Reporting Adverse Toxicological effects	Author Not Reporting Any Adverse Toxicological effects	Comments
		**3.2.1 Acute toxicity of aspartame**			
E46, 1973	rN	3.2.1.1 page 56		No remarkable motor or behavioural activities.<br>No mortalities in the experimental period (7 days)	
		Appendix F page 202		Motor and behavioural activities unremarkable.	
E84, 1974	rN	3.2.1.1 page 57	No, but inflammation was observed in the kidneys and endocardium	Physical, opthalmoscopic and body weight changes were unremarkable. No compound-related changes were observed at the gross or microscopic level, organ weights and organ to body weight ratios were unchanged.	Inflammation in kidneys and endocardium assumed, by authors, to be a result of an infection; ie not an adverse effect of aspartame
		Appendix F page 202	Phlebitis at site of implantation; kidney and endocardium	Physical, opthalmoscopic, gross or microscopic parameters unremarkable.	
E85, 1974	rN	3.2.1.1 page 57		All animals survived during the observation period (72 hours). Electrocardiogram patterns were unremarkable. No statistically significant changes were observed in the haematology, clinical chemistry and urinalysis parameters evaluated. Post-mortem gross and microscopic changes were unremarkable.	
		Appendix F page 202		Physical and biochemical parameters, ECC unremarkable	
		**3.2.2 Short-term and sub-chronic toxicity of aspartame**			

E2, 1972	rN	3.2.2.1 page 57	Intestinal mucosa from the high-dose treated mice was heavily coated with a clear, moderately viscous fluid	"No statistically significant difference in body weight was observed between treated and control animals. No adverse clinical conditions were noted and 100% survival was reported." (p 57)	5 mice per gender per dose group for 4 weeks. Implicitly, the wording acknowledged that there were 'effects' but discounted them as they were not 'unequivocal'. With only 5 animals per gender per dose group, the absence of a 'statistically significant' finding is not a reliable indicator of the absence of an adverse effect.
		Appendix G page 205	Clear, viscous fluid coating intestinal mucosa in treated rats.	"100% survival. No **unequivocal** effect on body weight….No adverse physical/behavioural changes; no treatment related pathological changes except for intestinal mucosa." (emphasis added)	Only the controls and those from the high dose group were examined.
E3, 1972	uP	3.2.2.1 page 57	Significant decrease in feed consumption. Intestinal mucosa from the treated rats was heavily coated with a clear, moderately viscous liquid.	No consistent statistically significant effect in body weight was observed. No adverse clinical conditions were reported and 100% survival was reported. No treatment related histopathological changes were reported from the high dose groups.	With only 5 rats per gender per dose group, the absence of a **consistent statistically significant** finding is not a reliable indicator of the absence of an adverse effect. Implicitly, the wording acknowledged that there were 'effects' but discounted them (Appendix G p 204) as they were not 'unequivocal'.
		Appendix G page 204	Decreased food consumption in high dose females, weeks 2-3. Clear, viscous fluid coating intestinal mucosa in treated rats.	"100% survival; no **unequivocal** effect on body weight or food consumption, except high dose females, wks 2-3." (emphasis added) No adverse physical/behavioural changes; no treatment related pathological changes except for intestinal mucosa.	"N.B. only examined 5 controls and all high dose animals; remaining animals were discarded." (p 204)
E20, 1969	rN & ELlow	3.2.2.1 page 57	In the high dose [125 mg/kg bw/day] males a significantly higher liver to body weight ratio was observed.	Survival was 100%. No effect on body weight and feed consumption. No effect on physical appearance or behaviour. No treatment related changes in haematology or urinanalysis. Organ weights were unaltered except in the high dose males. No histopathological finding except bile duct hyperplasia/pericholangitis in all groups.	The Panel discounted elevated liver weights and blood sugar levels. With only 10 animals per gender per dose group, of which only 5 examined after sacrifice) per group little reliance can be placed on its negative findings.

		Appendix G page 204	Increased liver/body weight ratio in high dose males. Increased terminal blood sugar at high dose.	No signs of systemic toxicity or pharmacological effects. No treatment related changes in haematology or urinanalysis. Apart from blood sugar level in high dose group there were no other significant changes in clinical chemistry. Organ weights were unaltered except in the high dose males. No histopathological finding except bile duct hyperplasia/pericholangitis in all groups.	But 'high dose' level was only 125 mg/kg bw/day. Changes were discounted as being neither **systemic** or **pharmacological**, which was implicitly setting a very high hurdle.
E21, 1969	rN	3.2.2.1 page 58		"...generally normal in appearance and behaviour throughout the study...no consistent effects upon organ weights were recorded and no consistent changes in haematological and clinical chemistry parameters, gross pathology and histopathology were observed."	The text implies there were 'findings' but they were not 'consistent'. **But this study used only 2 dogs per group, and lasted only 8 weeks.** The implicit assumption seems to have been that effects on only half of the dogs should not be deemed 'consistent. By in effect deeming this study to be a reliable negative, despite using only two dogs per group, the implicit hurdle of acceptability was ridiculously low.
		Appendix G page 204		"100 % survival" "No consistent haematology, clinical chemistry or urinalysis findings; no consistent ocular findings; no gross pathology; no consistent effects on organ weights; no cytopathological changes."	

Abhilash et al 2011	uP	3.2.2.2 page 58	"...a significant serum increases in activities of alanine aminotransferase, aspartate amino-transferase, alkaline phosphatase and gammaglutamyl transferase...a significant serum increases in activities of alanine aminotransferase, aspartate amino-transferase, alkaline phosphatase and gammaglutamyl transferase...The levels of GSH were significantly decreased...Histopathological examination revealed leukocyte infiltration...mild vascular congestion".		Panel stated "...that only six rats were used per group and that the exposure was not long term but only 6 months." But elsewhere, eg E 21, the Panel deemed as reliable shorter tests with fewer animals showing no adverse effects. Imperfections in the study were listed, and the authors quoted as saying larger studies were needed to confirm their findings. The Panel discounted the findings of 'significant changes' as if they could be unproblematically deemed as toxicologically insignificant. If they had taken the findings seriously, they should at least have called for longer-term tests on larger numbers of animals.
Abhilash et al 2013	uP	3.2.2.2 page 58	Yes "...a significant serum increases in activities of alanine aminotransferase, aspartate amino-transferase, alkaline phosphatase and gammaglutamyl transferase." "The levels of GSH were significantly decreased" "mild vascular congestion".		Panel stated "...that only six rats were used per group and that the exposure was not long term but only 6 months." But elsewhere, eg E21, the Panel deemed as reliable shorter tests with fewer animals showing no adverse effects. The Panel cited imperfections in the study, and cited the authors arguing that larger follow-up studies were required to confirm their findings. Panel discounted all the findings, but without calling for follow-up studies.
		**3.2.3. Genotoxicity of aspartame**			
E97 1978	rN	3.2.3.1 page 59		"Aspartame was not mutagenic in this [salmonella] test system"	
		Appendix H page 207			Salmonella test methods described as non-GLP but still accepted by Panel as reliably negative.
E101 1978	rN	3.2.3.1 page 59		"Aspartame was not mutagenic in this [salmonella] test system"	
		Appendix H page 207			Salmonella test methods described as non-GLP but still accepted by Panel as reliably negative. Also tester strains bearing AT mutation not used.

E81 1974	uN & Cont	3.2.3.1 page 59		"...the host-mediated assay revealed no evidence for mutagenicity"	"The Panel noted some discrepancies in description of doses in different sections of the report and that the test system employed has not received further validation and is presently considered obsolete and therefore, the results of the study were not included in the assessment." **But which other test systems have been 'validated', and what is to count as validation?**
	Cont	Appendix H page 210		Yes	Host mediated assay in mice. Methods thought sufficiently robust!
E40 1973	rN	3.2.3.1 page 59		The parameters analysed: paternal growth, maternal pregnancy rate, uterine and ovary examination data and incidence of foetal deaths were not affected by aspartame.	
		Appendix H page 209		Yes	
E41 1973	rN	3.2.3.1 page 59		The parameters analysed "paternal growth, maternal pregnancy rate, uterine and ovary examination data and incidence of foetal deaths" were not affected by aspartame	
		Appendix H page 209		Yes	
E43 1972	rN	3.2.3.1 page 59		"...did not increase the normal [chromosome] aberration frequencies compared to the control rats. The authors concluded that aspartame was not mutagenic."	"The Panel considered that the methods implemented were sufficiently robust to support the results reported, but considered the study limited since mitotic indices were not reported."
		Appendix H page 209		Yes	The panel commented that the mitotic index "should" be determined but overall thought the methods "sufficiently robust to support the results reported."
E12 1970	uN	3.2.3.1 page 60		"Aspartame was reported not to induce chromosome aberrations in bone marrow or spermatogonial cells"	Panel comments that "the dose levels applied were reported inconsistently" "The Panel considered that the reported results of this study were not supported by the outcome of the methods applied"
		Appendix H page 210			Chromosome aberrations in bone marrow erythrocytes and spermatogonial cells. Inconsistent reporting of dose levels plus other experimental problems. "...methods implemented were thought not to be sufficiently robust to support the results reported."

E44 1972	uN & Cont	3.2.3.1 page 60		"No statistically significant effects on mutation frequency were noted in the treatment groups, as compared to the control.	Rats, host mediated assay. "The Panel noted that the test system employed has not received further validation and it is presently considered obsolete, and therefore the results of the study were not included in the assessment."
	Cont	Appendix H page 210			"...methods thought sufficiently robust"
NTP 2005	rN	3.2.3.2 page 60		"No mutagenicity was detected in strains TA98, TA100, TA1535, TA1537 or TA97" "The authors of the study judged the small increase in mutant colonies with 30% rat liver S9 as equivocal."	Salmonella test. Tester strain bearing AT mutation not used. Small increase with one strain, deemed equivocal by NTP authors and consequently was discounted.
		Appendix H page 207			
Rencuzogullari et al 2004	uN	3.2.3.2 page 60		"No mutagenicity was observed."	Salmonella test, major deviations from OECD guideline so "...methods implemented were not sufficiently robust to support the results reported".
		Appendix H page 208			"...methods were thought not to be sufficiently robust to support the results reported".
Bandyopadhyay et al 2008	uN	3.2.3.2 page 60		"reported aspartame to be negative"	Salmonella test. "The Panel considered that the methods implemented were not sufficiently robust to support the results reported, due to major deviations from the OECD guideline"
Jeffrey and Williams 2000	rN	3.2.3.2 page 60		Aspartame was found to be negative in this assay"	rat hepatocyte/DNA repair assay.
		Appendix H page 208			
Rencuzogullari et al 2004 CA	uP	3.2.3.2 page 60	Yes, chromosomal aberrations at high dose of 2000 microgrammes/Litre.		Human lymphocytes, 3 tests. Panel noted several flaws and overall: "...the Panel considered the experimental findings reported in this study of limited relevance for aspartame risk assessment."
Rencuzogullari et al 2004 MN	uP		Yes, micronuclei chromosomal damage at high dose of 2000 microgrammes/Litre.		
Rencuzogullari et al 2004 SCE	uN			Yes, SCE	
		Appendix H page 208	Yes, chromosomal aberrations.		"...methods implemented were thought not to be sufficiently robust to support the results reported."
			Yes micronuclei		
				Yes, SCE	

Durnev et al 1995	uN	3.2.3.2 page 61		"...aspartame did not induce any increase in the incidence of chromosomal aberrations."	CA in BME, mice, "Study poorly reported" no positive control, inadequate sampling "...the Panel considered that the methods implemented were not sufficiently robust to support the results reported."
		Appendix H page 211		Yes	
Mukhopadhyay et al 2000	uN	3.2.3.2 page 61		"...blend of the two sweeteners showed a negative outcome for chromosomal aberrations."	CA in mice, blend of sweeteners and no mitotic index determination, so not deemed relevant for evaluation of aspartame.
		Appendix H page 211		Yes	Blend of sweeteners, the panel though not relevant to aspartame
NTP 2005 micronucleus	rN	3.2.3.2 page 61		"No increase in the number of micronucleated polychromatic erythrocytes was observed."	Rat micronucleus test in BME.
		Appendix H page 211		Yes	Male rat micronucleus test in BME. Deviation from OECD guidelines - time and type of animal used. Panel thought "methods sufficiently robust to support the results."
NTP 2005 (p53-haploinsufficient)	uP in haploinsufficient, but rN in other mice strains	3.2.3.2 page 61	Yes, female p53-haploinsufficient	Yes, in males	PB Micronucleus test, transgenic mice, 9 months, p53 haploinsuffiicent "The Panel concluded that the findings were equivocal in the p53 transgenic strain (positive in female but not in male p53 haploinsufficient mice) but negative in the other two strains, and, overall, did not indicate a genotoxic potential for aspartame."
NTP 2005 (TG.AC hemizygous)	rN	3.2.3.2 page 61		Yes	PB micronucleus test, transgenic mice, 9 mths, TG.AC hemizygous
NTP 2005 (CDkn2A deficient)	rN	3.2.3.2 page 61		Yes	PB Micronucleus test, transgenic mice, 9 months, Cdkn2a deficient
		Appendix H page 212		Yes	PB Micronucleus test, transgenic TG.AC hemizygous. Panel considered study robust but deviations from guidelines and no +ve control.
		Appendix H page 212	Yes in females	Yes in males	PB Micronucleus test, tg p53 haploinsufficient. Panel considered study robust but deviations from guidelines and no +ve control.
		Appendix H page 212-213		Yes	PB Micronucleus test, Tg Cdkn2a deficient. Panel considered study robust but deviations from guidelines and no +ve control.

Sasaki et al 2002	rN	3.2.3.2 page 62		"...did not induce any significant increases in DNA migration."	Comet using: "...a single dose of aspartame (2000 mg aspartame/kg bw) to mice (four male/group) and analysed the stomach, colon, liver, kidney, bladder, lung, brain, bone marrow. Aspartame did not induce any significant increases in DNA migration. Based on these results, the Panel considered that aspartame was not genotoxic in the organs assayed." **Even though this study administered only one dose, and only tested 4 mice, and the observed DNA migration was deemed not 'signifficant', so the panel deems this very weak study to be a reliable negative**
		Appendix H page 213		Yes	Comet assay. Appendix of Panel noted seven deviations from normal minimum requirements, ihncluding failure to include a posiotive control. Nonetheless, the panel asserted that: "The methods implemented were thought to be sufficiently robust to support the results reported." **Perhaps the most generous judgement in the entire report.**
Bandyopadhyay et al 2008	uP	3.2.3.2 page 62	Yes at highest dose used ie 35 mg aspartame/kg bw to mice.		Comet assay. n=4. ANS panel said: "...increases of DNA damage evaluated...were small and no historical control values were reported and used to exclude possible spontaneous biological fluctuations.. Therefore, the Panel considered that the methods implemented were not sufficiently robust to support the results reported in the study, and that no conclusion could be drawn from it....methods implemented were not sufficiently robust to support the results reported." **Ie despite fewer shortcomings than eg Sasaki 2002, the positive results were discounted and deemed as a false, or at any rate an unreliable, positive.**
		Appendix H page 213	Yes at high dose		Comet assay. Deviations from recommended protocol x 2 "...methods thought not to be sufficiently robust"
Kamath et al 2010	uP	3.2.3.2 page 62	Yes		Micronuclei tests (2 types) and CA in mice "...methods implemented were thought not to be sufficiently robust".
		Appendix H page 213/214	Yes		Micronuclei tests and CA in mice. Deviations from guidelines x 5 "...methods thought not to be sufficiently robust"
AlSuhaibani 2010 CA	uP	3.2.3.2 page 62	Yes,CA		Deviations from guidelines x 5 "...methods implemented were not sufficiently robust to support

AlSuhaibani 2010 SCE	uN			Yes, SCE	the results"
		Appendix H page 214	Yes, CA		"...the methods were thought not to be sufficiently robust"
Karikas et al 1998	uP	3.2.3.2 page 63	A non-covalent interaction of excess aspartame, aspartic acid and phenylalanine with calf thymus DNA.		"The Panel considered these findings...of minimal relevance for the evaluation of the genotoxic potential of aspartame."
Meier et al 1990	uP	3.2.3.2 page 63	Yes, the nitrosation products displayed an 'alkylating' activity *in vitro.*		Nitrosation is important because it has been implicated in some mechanisms of carcinogenesis."...the Panel noted the harsh conditions utilised for the *in vitro* nitrosation of substrates and considered the results of doubtful relevance for the assessment of the genotoxic risk posed by the dietary intake of aspartame"
Shephard et al 1993	uP	3.2.3.2 page 63	Yes, The nitrosation products of aspartame exhibited a direct mutagenic activity.		"...the Panel noted the harsh conditions utilised for the in vitro nitrosation of substrates and considered the results of doubtful relevance for the assessment of the genotoxic risk posed by the dietary intake of aspartame…"
		**3.2.4 Chronic toxicity and carcinogenicity of aspartame**			
E75 1974	uP	3.2.4.1 page 64	Yes, the relative weight of the heart to the thyroid was increased in females in both the high (4000mg/kg bw/day) and the low dose (1000mg/kg bw/day) groups. The panel refers to that as: "Random fluctuations reaching statistical significance were observed occasionally ...Body weight gain for the male mice at all dose levels was significantly lower than that for the male controls."		Mice, n=36/gp. NOAEL = 4000mg/kg bw/day, organ weights unaffected except for dose effects on heart/thyroid in females. "The authors concluded that aspartame, administered to the mouse for 104 weeks in the diet at dose levels of 1000, 2000 and 4000 mg/kg bw/day exhibited no adverse effects regarding survival rate, and that there was no evidence of an effect with respect to the incidence of neoplasms or with regard to non-neoplastic changes in any organ or tissue. The Panel agreed with this evaluation and identified a NOAEL for this study of 4000 mg/kg bw/day, the highest dose level tested."

E33-34 1973	ELhigh	3.2.4.1 page 64	Yes (> NOAEL) Females of the 8000 mg/kg bw/day group showed statistically significant lower survival rates. + other changes to lungs, ovaries, seminal vesicles, prostate, pancreas.		Statistically significant adverse effects discounted as occurring only at high dose level.
E70 1974	uP and ELhigh	3.2.4.1 page 65	Yes, growth rates for the exposed animals were comparable to growth rates of the controls except for the high dose (4000mg/kg bw/day) in males for which growth rates were significantly lower than for		Rats, n=40/gp. some effects, mostly dismissed by the authors. NOAEL = 4000mg/kg bw/day
E87 1973 (brains from E33-34 and E70)	rN	3.2.4.1 page 66		Yes, the panel concluded	Rats, intracranial neoplasm study. The tumours reported were random wrt dose and gender, so interpreted as not caused by aspartame.
Ishii et al 1981	uP and ELlow	3.2.4.1 page 68	Yes. "There was a dose-dependent depression of body weight gain at 2000 and 4000 mg/kg bw/day, and at 4000 mg/kg aspartame plus DKP (3:1) in males, and at all dose levels in females...a dose-related increase in focal mineralisation of the renal pelvis in both males and females (incidences in males: control, 1/57; 1000 mg aspartame/kg bw/day, 5/55; 2000 mg aspartame/kg bw/day, 10/60; 4000 mg aspartame/kg bw/day, 15/59; incidences in females: control, 16/59; 1000 mg aspartame/kg bw/day, 23/59; 2000 mg aspartame/kg bw/day, 30/59; 4000 mg aspartame/kg bw/day, 46/60)."		Rats, n=86/group, with evidence of dose-related changes. Focal mineralisation of the renal pelvis ws said by the panel to be of: "...minimal toxicological significance..." NOAEL = 4000mg/kg/bw/day. "…the Panel noted that the study provided information on the lack of toxicity of aspartame when administered in conjunction with DKP". But **some adverse effects were evident at doses below 4000mg/kg/bw/day, so that dose level should not have been deemed to be a NOAEL.**
E27 1972	uN	3.2.4.1 page 68	Nothing reported	Nothing reported	Hamsters, n=5 study, discounted for evaluation by panel because of: short duration, small numbers, infection - no (adverse) effects reported.

E35-36 1972	uN	3.2.4.1 page 68	Nothing reported	Nothing reported	Hamsters, n=5 study, discounted for evaluation by panel because of: short duration, small numbers, infection - no (adverse) effects reported.
E28 1972	uN	3.2.4.1 page 69	Yes, "...transient sporadic significant differences" in haematology and clinical chemistry findngs…significant increase in excretion of urinary pheylketones"		Dogs, n=5 per group. Transient sporadic differences (deemed not biologically meaningful) and "transient sporadic<br>significant differences were reported in several of the parameters." (p 69 lines 11-12) "A significant increase in excretion<br>of urinary phenylketones was observed in some high dose dogs at week 2, 4 and 26 of treatment, but<br>not at all other time intervals." NOAEL deemed = 4000mg/kg bw/day.
E86 1973	rN	3.2.4.1 page 69			Dogs, brain analysis of E28 as above. Deemed reliable negative despite just n=5.
E32 1972 = Waisman study	uP	3.2.4.1 page 69	Yes, *grand mal* seizures in lab monkeys.		The notorious Waisman monkey study. (see eg http://www.dldewey.com/columns/asparstu.htm and https://dash.harvard.edu/bitstream/handle/1/8846759/Nill,_Ashley_-_The_History_of_Aspartame.html?sequence=6 . 7 monkeys, 1-2 per group. *Shigella* and seizures! Rejected by Searle on the basis of *Shigella* infection. The Panel concluded that the study provided insuffficent information to conclude on the chronic effects of aspartame in monkeys. But no grounds for supposing Shingella could cause grand mal seizures. John Olney and James Turner both judged that sufficient information could be drawn from this study to indicate a risk that aspartame might cauise seizures in some vulnerable people.
Soffritti et al 2006	uP	3.2.4.2 page 70	Yes, increased numbers of various types of cancers.		Rats. N = 100-150 per dose gropup. The Panel used conclusions of previous evaluations of these studies and other ERF studies to conclude that "...methodological concerns…would apply to…the aspartame studies".

Soffritti et al 2007	uP	3.2.4.2 page 70	Yes, dose related increases in various types of cancers.		Rats, n = 70-95 per dose group. The Panel used conclusions of previous evaluations of these studies and other ERF studies to conclude that "...methodological concerns…would apply to…the aspartame studies".
Soffritti et al 2010	uP	3.2.4.2 page 71	Yes, dose related increases in various types of cancers.		Mice, n = 62-122 per dose group. The panel concluded (based on previous evaluations as well as this one) "...that the results of the studies..do not provide evidence for a carcinogenic effect of apartame in mice." BUT while they do not provide proof they certainly provide evidence of carcinogenicity in mice.
NTP 2005	rN - counted above	3.2.4.2 page 71		Yes	Study already listed. n=15/group. Transgenic, recorded again as looking for neoplasms.
		**3.2.5 Reproductive and developmental toxicity of aspartame**			
E11 1971	uP and ELlow	3.2.5.1.1 page 72	Yes. "...the body weights at the end of weaning of both sexes at the high dose (4000 mg/kg bw/day) level to be statistically significantly lower... than those of the controls."		Rats. NOAEL = 2000mg/kg bw/day, but not reported to current standards. Panel assessment of reliability - y (when used with other studies - E10, E47, E48). No - taken on its own. But many 'negative studies' not reported to current standards were deemed reliable.
		Appendix I page 227	Yes		
E9 1972	uP and ELlow	3.2.5.1.1 page 72	Yes, kidney - histological "...changes were treatment-related but of a transient nature."		Rats. F2 generation from above study, looking at haem and biochemical. changes transient (so deemed not adverse?). NOAEL = 2000mg/kg bw/day (implies ADI ≤ 20mg/kg bw/day). No reason given why 'transient' effects are not deemed adverse; nor why NOAEL is not 2000.
		Appendix I page 227	Yes.		Transient effects, not observed in other group of pups.
E10 1972	uP	3.2.5.1.1 page 72	Yes. "Female body weights were slightly but significantly decreased in the high dose group (fed 4000 mg/kg bw/day)."		Rats. N=12 males, 40 females. significant decrease in weight! NOAEL = 4000mg/kg bw/day "...actual ingested doses ranged from 4100 mg/kg bw/day during gestation to 5900 mg/kg bw/day during the last 14 days of lactation..." Statistical significance even in small sample.

		Appendix I page 227, column 6	Yes		In high dose group (4000 mg/kg bw/day) body weight "...suppression and smaller size of pups at weaning (statistically significant)."
E39 1973	uP and ELlow	3.2.5.1.1 page 73	Yes. "The number of viable pups per litter at birth and pup survival until weaning was significantly decreased in the high dose group" (intended 4000 mg/kg bw/day) "During lactation, maternal body weight..in the high dose group... mean body weight was significantly lower at postpartum day 21 (approximately 12 %)."		Rats. n=30. NOAEL = 2000 mg/kg bw/day during gestation, 3500 mg/kg bw/day during lactation. Panel noted that "...health status of all the animals in the study might have been compromised..." But: "According to the authors,the NOAEL in this study was 2000 mg/kg bw/day [which implies an ADI ≤ 20mg/kg bw/day]. "The Panel agreed with the author's conclusion on the NOAEL but noted that, considering the poor survival of control pups, the health status of all the animals in the study might have been compromised." Text said nothing about survival of control pups.
		Appendix I page 227-8	Y		"Number of viable pups per litter at birth and at weaning decreased (statistically significant)"
E88 1975	uP	3.2.5.1.1 page 73	Yes. "...two abortions reported were spontaneous..."		Monkeys. n=8, dose levels up to 3800 mg/kg bw/day, but treated as unreliable because incomplete data, no information regarding reproductive status and other factors.
E89 1975	rN	3.2.5.1.2 page 73		Yes.	Mice. n=36/gp. NOAEL = 5700mg/kg bw/day
		Appendix I page 227		Yes.	NOAEL = 4000mg/kg bw/day
E47 1973	uP, Cont and ELlow	3.2.5.1.2 page 74	Yes. High dose group, effects on various weight parameters and "...incomplete eyelid opening..." and "...body weight suppression of the pups at weaning (at postpartum day 21) in females at both low" (intended 2000mg/kg bw/day) "and high doses" (intended 4000mg/kg bw/day).		Rats. n=24/gp. High dose effects. NOAEL = 2500 mg/kg bw/day (gestation) and 3600 mg/kg bw/day (lactation). Anomaly - inconsistency betweeen panel suggested NOAEL and results.
	Cont	Appendix I page 228	Yes.		NOAEL = 2000 mg/kg bw/day

E48 1973	uP and Cont	3.2.5.1.2 page 74	Yes, high dose group (various weight parameters) and "...body weight suppression of the pups at weaning (at postpartum day 21) in females at both low and high doses" (doses as above)		Rats. n = 36/gp. high dose effects. NOAEL = 1800 mg/kg bw/day (gestation) and 3700 mg/kg bw/day (lactation). No reason given why NOAEL was not specified as 1800 mg/kg bw/day.
	Cont	Appendix I page 228	Yes.		NOAEL = 2000 mg/kg bw/day. No reason given why NOAEL is not 2000 or 1800 mg/kg bw/day.
E49 1973	uP	3.2.5.1.2 page 74	Yes. "...a significant decrease in body weight in the aspartame, L-phenylalanine and L-phenylalanine + L-aspartic acid groups…"		Rats. n= 30/gp. "The authors of the study concluded that L-phenylalanine on its own or in combination with aspartic acid decreased maternal and pup body weight, which reproduced the observed effects of aspartame on these endpoints. The Panel agreed with the author's conclusion but noted the poor survival of control pups." Significant adverse effects discounted, but reasons not given.
E5 1970	uP and Cont	3.2.5.1.2 page 74		Yes.	Rats. n= 30/gp. Food consumption in high dose (4000mg/kg bw/day) group decreased at beginning of treatment but recovered to control levels. NOAEL = 4000mg/kg bw/day.
	Cont	Appendix I page 227	Yes, at high dose, 4000mg/kg bw/day.	y	15% decrease in food consumption at high dose mentioned as an adverse effect. So why was that dose portrayed as a NOAEL?
E53 1973	uP, Cont, ELlow	3.2.5.1.2 page 75		y?	Rabbits.
	Cont	Appendix I page 227	Yes, at the high dose.		NOAEL = 2000 mg/kg bw/day, abortions in high dose group, but that group did not receive 2000 mg/kg bw/day but less!

E54 1974	uP and ELlow	3.2.5.1.2 page 75	Yes, "…a significant decrease in fetal body weight and skeletal anomalies..."		Rabbits. "…the Panel concluded from these observations that the developmental effects on body weight and skeletal development reported in the aspartame feeding studies may be caused by the significant depression of feed consumption in the high dose group." Although: "The actual aspartame doses were reported to be 1880 and 1870 mg/kg bw/day for the intended 2000 and 4000 mg/kg bw/day groups, respectively. The Panel noted that a significant decrease in fetal body weight and skeletal anomalies were reported for the 4000 mg/kg bw/day group but not for the 2000 mg/kg bw/day group even though both groups received the same dose of aspartame based on feed intake." **But if they had the same intake then the panel's reasoning is flawed!**
		Appendix I page 227	Y (with just 16 animals per group)		NOAEL = 2000 mg/kg bw/day
E55 1973	uP and ELlow	3.2.5.1.2 page 75			Rabbits. No comments either way
		Appendix I page 229	Yes, at the high dose.		"Feed consumption decreased by up to 29% in the high dose group." Nonetheless the panel portrayed 4000 mg/kg/bw as a NOAEL, **but that group actually received ~ 1160 mg/kg.**
E62 1973	uP	3.2.5.1.2 page 75			Rabbits.
		Appendix I page 229	Yes, at high dose, with 3 fetuses of one litter open eye (considered by the authors as minor malformations).	y	Minor malformations at 2000mg/kg bw/day. Nonetheless ANS panel says: NOAEL = 2000mg/kg bw/day. **Text in main body and in appendix conflict**

E63, 1973	uP, Cont and ELlow;	3.2.5.1.2 page 75	Yes, “There were no deaths in the pair-fed control group, two deaths in the 1400 mg/kg bw group and three deaths in the 2400 mg/kg bw group. One female in the high dose group had an early delivery. Conception rates were 96, 81 and 77 % in the control, 1400 and 2400 mg/kg bw groups, respectively.”		Rabbits. n=26/gp. “There were no deaths in the pair-fed control group, two deaths in the 1400 mg/kg bw group and three deaths in the 2400 mg/kg bw group. One female in the high dose group had an early delivery. Conception rates were 96, 81 and 77 % in the control, 1400 and 2400 mg/kg bw groups, respectively.”
	Cont	Appendix I page 229			No comment, but states: NOAEL=2400 mg/kg bw/day. **Text in main body and in appendix conflict with each other.**
E51 1973	uP, Cont and ELlow	3.2.5.1.2 page 76	Yes. The administration of aspartame was associated with depression of feed consumption by up to 40%.	"The authors concluded that no embryotoxic or teratogenic effects were observed."	Rabbits. n=36/gp The administration of aspartame was associated with depression of feed consumption by up to 40%. "The study was confounded by poor health of the animals and the gavage technique issues. "
	Cont	Appendix I page 230	Yes. Doses of 0, 2000 mg/kg bw/day “GD 6-18; pregnant animals: 5,11 Deaths: 4,9 , Abortions: 1,2"	Yes	“GD 6-18; pregnant animals: 5,11 Deaths: 4,9 , Abortions: 1,2” Doubled death rates and abortions ignored. NOAEL = 2000 mg/kg bw/day although “Study confounded by poor health and gastric intubation technique issues; high maternal mortality; depression of feed consumption by 40% compensated by pairfeeding of controls... Very low pregnancy rate. Authors mention infectious pulmonary disease and dosing errors. No malformations.” **But authors conclusions did not reflect the data.**
E52 1973	uP, Cont and ELlow	3.2.5.1.2 page 76		"The authors concluded that no embryotoxic or teratogenic effects were observed."	Rabbits. n=72/gp "...maternal mortality due to poor health and the gavage technique issues..."

	Cont	Appendix I page 230	Yes. 0,2000 mg/kg bw/day "...pregnancy rate decreased in high dose group; depression of feed consumption by up to 62% compensated by pair-feeding...”		NOAEL = 2000 mg/kg bw/day although: “Study confounded by poor health and gastric intubation technique issues; pregnancy rate decreased in high dose group; depression of feed consumption by up to 62% compensated by pair-feeding.”
E79 1974	uP, Cont and ELlow	3.2.5.1.2 page 76		"The authors concluded that no embryotoxic or teratogenic effects were observed."	Rabbits. 37 received dose of 750 mg/kg bw/day and 95 had 2000 mg/kg bw/day. “The study was confounded by poor health of the animals and the misdosing. As a result, maternal mortality was high. The administration of aspartame was associated with decreased feed consumption by up to 36 %. The control animals were pair-fed to match the aspartame-treated animals with the lowest feed intake. However, especially aspartame-treated animals with a very restricted feed consumption died and the aspartame-treated animals with a more normal food consumption survived. As a result, the food consumption in the pair-fed control animals was noted to be lower than the food consumption of the aspartame-treated animals. The authors concluded that no embryotoxic or teratogenic effects were observed. However, the Panel considered the study not to be adequate to reach such a conclusion.”
	Cont	Appendix I page 230	Y “Major malformations in 7 fetuses of high dose group” – **findings not mentioned on page 76**		NOAEL = 750 mg/kg bw/day

E90 1975	uP and ELlow	3.2.5.1.2 page 76	Yes. "A number of animals died spontaneously during the study… mainly due to misdosing. … No abortions were detected in the control, mid dose and L-aspartic acid groups, two abortions in the low dose aspartame group and 24 abortions were observed in the high dose aspartame group (a significant increase compared to controls) and four in the L-phenylalanine group"		Rabbits."...contained a sufficient number of animals for the evaluation of developmental toxicity" high dose effect. NOAEL = 1000mg/kg bw/day. A number of animals died spontaneously during the study… mainly due to misdosing. … No abortions were detected in the control, mid dose and L-aspartic acid groups, two abortions in the low dose aspartame group and 24 abortions were observed in the high dose aspartame group (a significant increase compared to controls) and four in the L-phenylalanine group. .. Since the decrease in body weight started several days before (13 and 18 days) the abortions (28 days), the authors concluded that abortion was a consequence of significant and rapid body weight loss caused by decreased feed
		Appendix I page 231	Yes.		NOAEL = 1000mg/kg bw/day
Brunner et al 1979	ELhigh	3.2.5.2.1 page 78	Yes, at high doses, 5,000 mg/kg bw/day; 6000; 9,000. "Increased offspring mortality was observed in rats fed the highest aspartame dose and in the phenylalanine-exposed animals."		Rats, reproduction study, number of rats not stated. Adverse effects reported in original Brunner study, but "…the Panel agreed with Brunner et al" that the adverse effects only occurred at highest doses, but sample size was small so cannot provide reassurances at lower doses.
Lennon et al 1980	uN	3.2.5.2.1 page 78		"There were no differences noted in the number of rats that became pregnant."	Study funded by US FDA. Rats, 6 per group, *post coital* fertility, **followed for just 7 days**. "The Panel noted that the number of animals used in these studies was small."
Lennon et al 1980	uN	3.2.5.2.1 page 78		"...no differences were reported in implantation and regression of corpora lutea."	Hamsters, 5 per group, *post coital* fertility, **followed for just 7 days**. "The Panel noted that the number of animals used in these studies was small."
Mahalik and Gautieri 1984	uP	3.2.5.2.2 page 79	Yes, "...the achievement age for visual placing was significantly delayed, in a dose-dependent manner, in both groups of treated animals."	"There were no significant differences between control and treated animals in negative geotaxis, surface or air righting."	Mice, number not reported. "The Panel noted that uncontrolled litter size and pup weight were not taken into consideration and performance of the offspring was assessed only the last day of achievement for the entire litter."

McAnulty et al 1989	uP and ELlow	3.2.5.2.2 page 79	Yes. "Time of eye opening was statistically significantly later than control at the lowest and highest doses (14.3 ± 0.15 for both doses vs. 14.8 ± 0.15 in controls), as well as the development of the visual placing which was statistically significantly lower than control at the lowest dose of 500 mg aspartame/kg bw/day (18.8 0.28 vs. 20.5 ± 0.40 in controls)."	"...*in utero* exposure to aspartame in CF-1 mice did not affect the physical and functional development of the visual system of the pups."	Mice, 20/group. Significant changes, but authors concluded not biologically meaningful, and the panel agreed. "Time of eye opening was statistically significantly later than control at the lowest and highest doses (14.3 ± 0.15 for both doses vs. 14.8 ± 0.15 in controls), as well as the development of the visual placing which was statistically significantly lower than control at the lowest dose of 500 mg aspartame/kg bw/day (18.8 0.28 vs. 20.5 ± 0.40 in controls). The authors considered these findings as isolated points that may vary in either direction. They were not dose-related and as such, the results were not considered to be biologically meaningful. " **Discounted as non-monotonic dose-response relationship.**
Holder 1989	rN	3.2.5.2.2 page 79/81		"...findings indicated to the authors that spatial memories, as well as motor and visual components of these tasks were not affected by perinatal exposure to aspartame."	Rats, n=10/gp. "The authors concluded that exposure in utero and later directly of the pups did not affect reflex development, morphological development and spatial memory. The Panel agreed with the conclusion of the authors."
NTP-CERHR Report 2003	rN & ELlow	3.2.5.2.2 page 79		Yes.	Monkeys. NOAEL = 2500-2700 mg/kg bw/day (highest dose tested). not known how many.
Collison et al 2012a	uP	3.2.5.2.2 page 80	Yes. "The authors concluded that aspartame exposure might promote hyperglycaemia and insulin intolerance, and MSG might interact with aspartame to impair further glucose homeostasis."		Mice, n= 12-18/group. MSG study too. Panel noted problems with mouse strain used and no dose response assessed, and other reasons for negating outcome including data from Anton et al 2010 which were "..short term preliminary interventional trials.."

Collison et al 2012b	uP	3.2.5.2.2 page 80	Yes. The authors of the study concluded that lifetime exposure to aspartame, commencing in utero, might affect spatial cognition and glucose homeostasis in Cont7BL/6J mice, particularly in males.		Mice, n=12-18/gp. Panel noted problems with the method reporting, no dose response assessed and statistical procedures. The Panel noted that the selection method of pups for several tests was not clearly reported by Collison et al and that only one dose level was used thus rendering any assessment of dose-response relationship impossible. The Panel noted that the findings in mice reported by Collison et al (2012 b) might not apply to other species, since in a large study on Sprague-Dawley rats (Holder, 1989) performances on radial-maze and milk maze was similar for rat pups given aspartame at doses from 14 to 1614 mg/kg bw/day or phenylalanine at a dose of 835 mg/kg bw/day compared to controls.
Lennon et al 1980	ELhigh	3.2.5.2.2 page 81	Yes "Feed consumption and body weights were significantly lower in dams fed 7.5 and 14 % aspartame diets on day one and throughout the experiment (e.g. body weight reduction by up to 65%). " (9110 and 8830 mg/kg bw/day)	"...no effects...at doses up to 7120mg/kg bw/day on feed consumption, dam and pup body weight, pup survival and mammary gland histology"	Rats, n=60. High dose effects. NOAEL = 7120 mg/kg bw/day.
Ranney et al 1975	uP	3.2.5.2.2 pages 81-82	Significant increases to "Maternal plasma phenylalanine and tyrosine levels" and "Fetal plasma tyrosine was significantly higher in aspartame-fed animals compared to controls..." and other changes too.	Yes	Rabbits, n=30. Significant increases to "Maternal plasma phenylalanine and tyrosine levels" and "Fetal plasma tyrosine was significantly higher in aspartame-fed animals levels" and other changes but the ratios between concentrations changed little, so level changes were discounted by the authors "The authors concluded that the treatment of pregnant rabbits with a high dose of aspartame did not affect the transport of phenylalanine and tyrosine across the placental membrane since the ratios of fetal/maternal plasma amino acid concentrations were unaffected by the treatment."
		**3.2.6 Other studies on aspartame**			

E94 year not reported	uP	3.2.6.1 page 82	Yes. Original typescript of report stated p. 5: "Major lesions…were largely confined to mid-line structures, namely the hypothalmic arcuate nucleus, the subfornical organ and the area postrema."		Mice. No results described in report. "The Panel however noted that the design of the study was not adequately described and no control group was included, and, therefore, this study was considered not relevant for the overall risk assessment."
Reynolds et al 1976	uP	3.2.6.1 page 82	Yes		Reported by the ANS panel as a study on mice. No results were described in the ANS's report. The Panel "...noted the absence of control animals in the study and therefore did not take this study into consideration." But the paper was mis-reported by ANS panel. It was not simply a study of aspartame with mice, it tested both aspartame and msg in both neonatal mice and infant macaque monkeys. In effect the MSG and the aspartame groups were used as if controls in relation to each other, as the purpose of the study was primarily comparative. Moreover the authors said, when comparing the effects of aspartame with those caused by consuming msg: "The lesion encountered at 2 g/kg of aspartame is quite similar to what is seen in a neonatal mouse treated at 0.5 g/kg MSG..." (p 476) G D Searle funded this study.
E14 1972	uP, ELhigh	3.2.6.1 page 82	Yes, at the high dose. "For the high dose groups (5 % phenylalanine and 9 % aspartame), ***statistically significantly*** [emphasis added] [in groups of 32 animals] impaired learning performances were reported (i.e. conditioned or nondiscriminated avoidance responses)." (6100mg/kg bw/day (male); 6900mg/kg bw/day (female))	Yes, at lower dose.	The ANS panel discounted evidence of adverse effects as they were only reported at doses > 4000 mg/kg bw/day.

Beck et al 2002	uP	3.2.6.1 page 83	Yes. "...aspartame treated rats gained less body weight than controls" ($p<0.02$)...the level of hypothalamic neuropeptide Y in the arcuate nucleus (but not in other parts of the brain) was significantly lower in aspartame-treated rats than in controls by 23.2 % ($p < 0.02$)."		Rats, significant decrease in levels of neuropeptide-Y but authors not able "...to elucidate if the observed effects are physiological or treatment-related." Further more: "The author reported that the reasons for the neuropeptide-Y decrease were not clear and further analysis of other neuropeptides in the arcuate nucleus plus further histological controls will contribute to elucidate if the observed effects are physiological or treatment-related. The panel agreed with these observations." The ANS panel is unclear if this effect is a physiological or toxicological effect.
Christian et al 2004	uP	3.2.6.1 page 83	Yes. "From day 90 onward, latencies of the aspartame group increased which the authors interpreted as a sign of memory loss."		Rats, n=12, testing learning and memory. Panel dismissed findings because of type of reward used; but did not indicate which other type of reward could have been suitable. "The Panel concluded that this study is insufficient to ascertain an effect of aspartame on memory functions."
Puica et al 2008	uP	3.2.6.1 page 83	Ultrastructural damage, described as "...selective degeneration of all subcellular neurons ultrastructures both in CA1 pyramidal neurons of the hippocampus and ventromedial area of the hypothalamus'…" was reported in rabbits.		Rabbits, brain structure - dismissed because of experimental, reporting and analytical problems "...authors conclude that juvenile rats and rabbits are particularly susceptible to neurotoxic effects induced by aspartame." However, the Panel noted that the interpretation of these studies was not possible because of the lack of experimental details, the absence of appropriate control animals and of statistical analysis of the data." 2 separate studies, reported together by the Panel.
Puica et al 2009	uP	3.2.6.1 page 83	Ultrastructural damage, described as "...selective degeneration of all subcellular neurons ultrastructures both in CA1 pyramidal neurons of the hippocampus and ventromedial area of the hypothalamus..." was reported in rats.		Rats, brain structure - dismissed because of experimental, reporting and analytical problems "...authors conclude that juvenile rats and rabbits are particularly susceptible to neurotoxic effects induced by aspartame." However, the Panel noted that the interpretation of these studies was not possible because of the lack of experimental details, the absence of appropriate control animals and of statistical analysis of the data."
E104 1979	rN	3.2.6.1 page 83		Yes.	Monkeys, n=2-6/gp. These 3 studies are grouped together and "...the panel noted that the design of the study is inappropriate to evaluate effectively any potential neurotoxic effects of aspartame."
Reynolds et al 1980	rN	3.2.6.1 page 83		Yes	

E105 year not reported	rN	3.2.6.1 page 83		Yes	
Magnuson 2007	rN	3.2.6.1 page 84		Yes	Three reviews of the neurotoxicity of aspartame concluded that the data obtained from the extensive investigations of the potential neurotoxic effects due to aspartame consumption did not support the hypothesis that aspartame present in the human diet would cause any impairment of the neuronal function, learning or behaviour. "The Panel agreed with their conclusions." The guidelines have criteria for inclusion/exclusion which state "Types of studies that were considered within the criteria for inclusion in the selection process: a) Experimental studies b) Epidemiological studies in humans, c) Case reports supported by medical evidence." But unclear about inclusion of reviews. One question is whether other reviews should also have been ncluded? **Citing SCF 2002 and EFSA 2010 are examples of 'institutional inertia', ie an institution never criticising its prior judgements or those of its predecessors.**
EFSA 2010 Review	rN	3.2.6.1 page 84		Yes	EFSA agreed with EFSA, an example of institutional inertia.
SCF 2002 Review	rN	3.2.6.1 page 84		Yes	EFSA agreed with its predecessor, an example of institutional inertia.
E15 1972	uN	3.2.6.2 page 84		"...no acute effect on hepatic cytochrome P450 (CYP)-mediated xenobiotic metabolism..."	Rats. "The Panel noted that the findings have minimal relevance for human risk assessment." **But no reasons were given.**
Tutelyan et al 1990	uP	3.2.6.2 page 84	Yes, small effects on hepatic Phase 1 and Phase 2 metabolism in rodents after 45 days.		Rats. The Panel noted that the findings have minimal relevance for human risk assessment. **But no reasons were given.**

Vences-Mejia et al 2006	uP	3.2.6.2 page 84	Yes, "…bands corresponding to CYPs 1A1, 1A2, 2B, 3A2 were detectable in the cerebrum and cerebellum samples brain samples after aspartame treatment (but not in the control samples) and increases in all the enzyme activities were reported."		"The Panel noted that the findings have minimal relevance for human risk assessment." **But no reasons were given.**
Alleva et al 2011	uP	3.2.6.3 page 84	Yes. "Transient increases in the inflammatory cytokine IL-6 and the growth factor VEGF-A were also observed in the HUVEC cells following treatment with aspartame. This increase coincided with a temporary induction of ROS in HUVEC cells but not in IMR-90 fibroblasts thus concluding that generation of ROS is related to the target."		*In vitro* model. The panel concluded the study was not relevant; no controls in either part. The Panel noted that production of ROS could not be attributed to specific cell types but rather a general phenomenon. Furthermore, the authors did not evaluate the fate of aspartame in culture medium ascertaining whether it was hydrolysed to its usual metabolites or remained intact. For induction of angiogenesis, no positive and negative controls were reported. Therefore, the findings reported might be ascribed specifically to the conditions of the study. For these reasons, the Panel considered that this study was not relevant for the risk assessment of aspartame.
Haque and Mozaffar 1993	uN	3.2.6.3 page 84		The study authors concluded that aspartame does not affect AChE activity.	Mice, n=42/group. Effects on acetylcholine esterase (AChe). "The Panel noted that there was an error in authors' calculation of aspartame daily consumption dose, because applying EFSA's conversion factor (EFSA Scientific Committee, 2012) gives a daily dose of 2150 and 1690 mg/kg bw for females and for males respectively."

Simintzi et al 2007a	uP	3.2.6.3 page 85	Yes, at high doses. "Mix 2 caused a significant reduction only of the AChE [acetylcholine esterase] activity from the frontal cortex" and of AChE pure enzyme after incubation with mix 3 and 4." (150 mg/kg bw (mix 3) and 200 mg/kg bw (mix 4).)		*In vitro* model. The panels says: "...the authors concluded that AChE activity could be reduced by aspartame metabolites after ingestion of very high amounts of aspartame. Metabolites derived from a realistic beverage consumption of aspartame does not affect AChE activity. The Panel, noting the unrealistic high doses of aspartame metabolites assumed to be present in CSF and **several assumptions and speculations** that were applied to explain the effect on AChE activity, considered that no conclusions can be drawn from these studies. The Panel concluded that these studies are of no relevance for aspartame risk assessment under realistic use conditions." (p 85) (emphasis added)
Simintzi et al 2007b	uP	3.2.6.3 page 85	Yes. "Mix 3 and 4 induced a similar reduction of AChE activity from hippocampal protein extract"		*In vitro* model, Ache, dismissed because of assumptions and speculations. Panel deemed this study of "no relevance" under "realistic use conditions"
Kim et al 2011	uP	3.2.6.3 page 86	Yes. Paper in *FdChemTox stated*: "After 12 days, 30% of zebrafish, which consumed aspartame and HCD, died with exhibiting swimming defects." p 2903: "In the absence of cholesterol, the aspartame group had an increase in the inflammatory response, which was correlated with increased infiltration of inflammatory cells and production of ROS in the liver and brain." ANS report stated: "No effect of aspartame on survival was observed."		Zebrafish. n=70. ANS Panel report stated: "No effect of aspartame on survival was observed." …but it did cause an 'inflammatory response' Kim JY, Seo J and Cho KH, 2011 stated: "Aspartame-fed zebrafish exhibit acute deaths with swimming defects and saccharin-fed zebrafish have elevation of cholesteryl ester transfer protein activity in hypercholesterolemia." *Food and Chemical Toxicology*, 49, 2899-2905. Original paper suggested that aspartame on its own did not show adverse effects, but had serious adverse effects when combined with high cholesterol diet. **Study mis-reported by the ANS panel.**

E1 1972	rN	3.2.6.3 page 86		"The compendia did not report any adverse effects."	Following a public call for data, preliminary investigations on a wide range of potential pharmacological and endocrine effects of aspartame, summarised in two data compendia (E1, 1972; E19, year not provided), were received.
E19 year not provided	rN	3.2.6.3 page 86		"The compendia did not report any adverse effects.	
		**3.2.7 Human studies of aspartame**			
SCF 2002, Review	rN	3.2.7.1 p 86		"...that there was no evidence for adverse effects of aspartame in the human population."	"Epidemiological data on aspartame were previously reviewed by SCF (2002). The Panel considered and agreed with the conclusions of SCF." This item was cited above, and has already been counted as a 'uP'. The ANS panel's endorsement of the SCF assessment is a candidate for 'instutional inertia'.

Halldorsson et al 2010	uP	3.2.7.1 page 86	Yes. “Statistically significant trends were found in the risk of pre-term delivery with increasing consumption of artificially sweetened drinks (both carbonated and non-carbonated), but not for sugar-sweetened drinks."		Retrospective epidemiological study: 1996-2002. Limitations mentioned, replication suggested by authors. Panel agree with authors that replication wasrequired, implying that it agreed with authors that their study had major strengths (p 87, para 5, first line), and that there were no important flaws in the methods used. However, *panel speculated that risk estimates may have been inflated by residual confounding* (including by year of delivery). No account was taken of other dietary sources of methanol, and use of aspartame specifically was not distinguished from that of other artificial sweetener). Therefore, given these limitations, the Panel agreed with the authors who concluded that replication of their findings in another setting was warranted.” However, if the findings were treated as reliable the conclusion of the section and the report would have been conspicuously different, but the panel concluded: "Overall, currently available epidemiological data do not suggest that consumption of artificially sweetened soft drinks is a cause of pre-term delivery." (p 88) The panel did not set a temporary ADI contingent upon attempts to replicate the findings.

Englund-Ögge et al 2012	uP	3.2.7.1 page 87	Yes. Small elevations of risk [for pre-term delivery] were observed with higher consumption of artificially sweetened soft drinks, but after adjustment for covariates, these reached statistical significance only when categories of consumption were aggregated to four levels, and then the odds ratio for the highest category (≥ 1 serving/day) was only 1.11 (95 % CI 1.00-1.24) in comparison with non-consumption. but only far weaker than Halldorson (also		1999-2008 "Both Halldorsson et al. (2010) and Englund-Ögge et al. (2012) studies appear to have been well designed and conducted. Noting this, the Panel concluded that even at high levels of exposure to artificially sweetened soft drinks the risk of pre-term delivery is likely to be small, if any. **The observed associations could be a consequence of uncontrolled residual confounding**, and the inconsistencies in the patterns of association reinforce this uncertainty." The panel interprets Englund-Ögge et al as refuting Halldorsson et al and being a replication rather than the other way round, and acknowledging that they were overlapping studies. The panel did not set temporary ADI contingent upon attempts to replicate the findings.
La Vecchia 2013 - meta-analysis of Halldorsson et al (2010) and Englund-Ögge et al (2012)	uP	3.2.7.1 page 88	Yes, also sugar sweetened.		Meta analysis of above 2 studies, but elevated risk was evident with sugar sweetened drinks too! Panel said: "The analysis indicated similarly elevated risks of pre-term delivery with higher consumption both of sugar-sweetened and of artificially sweetened drinks. This lack of specificity in the associations again points to possible residual confounding. Currently available epidemiological data do not suggest that consumption of artificially sweetened soft drinks is a cause of pre-term delivery." (p 88)
Maslova et al 2013	uP	3.2.7.1.2 page 88	Yes, increased risk of asthma.		Asthma, "...weakly suggestive of hazard..." Limitations mentioned, further exploration suggested. "Because in epidemiological terms, the elevations of risk were only small and inconsistent, the findings from this study can only be considered weakly suggestive of hazard i.e. an association between the consumption of artificially sweetened beverages during pregnancy and the diagnosis of asthma or allergic rhinitis in children. Before a final conclusion can be reached with regard to aspartame, the findings need to be explored further with more detailed assessment of exposure to specific artificial sweeteners."

Hardell et al 2001	uP	3.2.7.1.3 page 89	Yes. "Non-significant elevations of risk were observed for consumption of such drinks in relation to brain tumours overall (OR 1.24, 95 % CI 0.72-2.14) and malignant brain tumours specifically (OR 1.70, 95 % CI 0.84-3.44). "Non-significant elevations of risk were observed for consumption of such drinks in relation to brain tumours..." with odds ratio higher than for any other parameter.		The ANS panel commented that: "The study had a high response rate, but was limited by its relatively small size, the basic assessment of exposure (low-calorie drinks were the only source of aspartame investigated in this study), and the potential for recall bias (because cases knew that they had a brain tumour) all of which could have led to spurious inflation of risk estimates." But the authors reported **a 24% increased risk** for regular consumption of artificially-sweetened beverages compared to non-consumers, which was very close to being staistically significanty. The fact that the odds ratio was lower than for other parameters is irrelevant, given that the authors chose to study factors strongly suspected to increase cancer risks. Moreover, the fact that the study only gathered data on the consumption of artificially-sweetened beverages, rather than all sources of aspartame intake, strengthens the evidence rather than weakens it.
Bunin et al 2005	uP	3.2.7.1.3 page 89	Yes: "….no significant elevations of risk.." although there was a significant trend before adjustment.		Study of 315 US children with specific types of brain tumours. Significant trend before adjustment. Limitations of study described that "...restrict the conclusions that can be drawn from this study."
Gallus et al 2006	uN	3.2.7.1.3 page 90		"...the results do not suggest a hazard for the cancers studied"	Case-control studies, n=7028 (9 cancers), on their own they provide only limited reassurance of safety.
Bosetti et al 2009	uN	3.2.7.1.3 page 90		The "...findings do not suggest a hazard"	Panel commented that because of limitations of study "...they provide only limited evidence of safety."
Andreatta et al 2008	uP	3.2.7.1.3 page 91	Yes, "…a positive association was found between long-term use of artificial sweeteners and risk of Urinary Tract Tumours."		Study referred to all artificial sweeteners, not specifically aspartame, "80% of both cases and controls who consumed artificial sweeteners, used saccharin and/or cyclamate. Thus, the study provided little information about possible risks from aspartame."
Lim et al 2006	rN	3.2.7.1.3 page 91		"The authors concluded that...aspartame consumption ...does not raise the risk of haematopoietic or brain malignancies."	Panel cited limitations of study but added: "Confounding is unlikely to have been a major problem."
Cabaniols et al 2011	uN	3.2.7.1.3 page 91		"There was no association with aspartame consumption"	Limitations of study noted. "...the non-positive finding provides little reassurance of an absence of hazard."

Schernhammer et al 2012	uP	3.2.7.1.3 page 92	Yes, at highest level of aspartame intake "significantly elevated relative risk of NHL (1.64, 95 % CI 1.17-2.29) and of multiple myeloma (3.36, 95 % CI 1.38-8.19) in men"	"...no corresponding elevations in risk in women. No clear association with leukaemia was apparent in either men or women."	The panel cited imitations of study. "...the authors proposed explanation for the differential associations in men and women is unconvincing...the positive findings can be given little weight."
E66 1973	rN	3.2.7.2 page 92		Yes.	n=2
		Appendix J page 232		Yes.	Case report
E110 1979	rN	3.2.7.2 page 92		Yes.	n=6. looking for 'Chinese restaurant syndrome'.
E23 1972	rN	3.2.7.3 page 92		Yes.	n=31-33. 6 weeks.
E24 1972	rN	3.2.7.3 page 93		Yes.	n=84. 6 weeks.
E60 1973	rN	3.2.7.3 page 93		Yes.	Follow up study of E23, for 21 weeks.
E61 1972	rN	3.2.7.3 page 93		Yes.	Children and adolescents for 13 weeks. Minor differences considered clinically trivial and non persistant.
E95 1977	rN	3.2.7.3 page 94		Yes.	Non-significant elevation of phe and tyr levels. 6 individuals receiving asp with MSG. This was more of a metabolic study than a toxicological one, but the panel treats it as a reliable toxicological negative.
		Appendix J page 233		Yes.	
Leon et al 1989	rN	3.2.7.3 page 94		Yes.	n=101
Porikos and Van Italie 1983	rN	3.2.7.3 page 94		Yes.	n=21, aspartame dose not specified. But this was not a toxicological test, and the sample was bizarre. The participants were drawn from people who were homeless and under-nourished.
E25 1972	rN	3.2.7.3 page 94		Yes.	65 people tested across 3 labs.
E67 1973	rN	3.2.7.3 page 95		Yes.	
		Appendix J page 238		Yes.	
E109 1978	rN	3.2.7.3 page 95		Yes.	PKU heterozygotes n=5, n=6 normal
E26 1972	rN	3.2.7.3 page 95		Yes.	2 boys, PKU homozygous
		Appendix J page 242		Yes.	
Krusei et al 1987	rN	3.2.7.4 page 96		"...no significant differences in any of the 39 cognitive and behavioural variables"	N=30, lower but not significantly different activity reported during aspartame challenges.
Wolrach et al 1984	rN	3.2.7.4 page 96		"...did not observe any significant effect of apartame on cognitive, attentitive or behavioural testing."	N=70

Shaywitz et al 1994a	rN	3.2.7.4 page 96		"The authors did not observe any significant effect of aspartame administration on cognitive, attentive or behavioural testing"	Children with ADD, n=15, changes to pheylalanine deemed 'acceptable'.
Roshon and Hagen 1989	rN	3.2.7.4 page 96		"...did not find any significant difference in locomotion, task orientation and learning"	N = 6 of each sex
Saravis et al 1990	rN	3.2.7.4 page 97		"...did not have a detrimental effect on learning, behaviour and mood in children."	Number not given
Lapierre et al 1990	rN	3.2.7.4 page 97		"No significant differences... were found in measures of sedation, hunger, headache, reaction-time, cognition or memory"	N=10, changes to pheylalanine levels deemed acceptable.
Ryan-Harshman et al 1987	rN	3.2.7.4 page 97		Neither phenylalanine nor aspartame altered mean energy intakes or macronutrient selection nor caused any behavioural effects.	N=13/group
Pivonka & Grunewald 1990	rN	3.2.7.4 page 97		"The only observed effect was increased sleepiness following the consumption of sugar-sweetened beverages."	N=120
Stokes et al 1991	rN	3.2.7.4 page 97		"...no detectable performance decrements were associated with the exposure to aspartame"	N=12
Stokes et al 1994	rN	3.2.7.4 page 97		"...no significantly impaired performance on flight-relevant cognitive tasks were observed..."	Apparent improvement in aviation-relevant cognitive task after aspartame
Walton et al 1993	uP	3.2.7.4 page 97	Yes. "…there was a significant difference in the number and severity of self-scored symptoms between aspartame and placebo in the patient [depressed] group."	"...there was no noted difference in the non-depressed volunteer group"	The methodology was a randomised double-blind cross-over challenge study, ie it was designed to use a 'gold standard' methodology. It aimed to test 40 patients with depression and 40 without. It was stopped after just 13 patients had been tested. Small numbers, but adverse reactions of 3 patients caused test to be stopped. Given the statistically significant evidence evidence generated by this study, and the uncertainties attending it, it is puzzling that the ANS panel failed to call for further studies; on this issue or on any other.

Spiers et al 1998	rN	3.2.7.4 page 98		"...no neuropsychologic, neurophysiologic and behavioural effects linked to aspartame consumption were observed."	
Panel noted limitations to all studies in Section 3.2.7.4 but concluded "...no evidence that aspartame affects behaviour or cognitive function in children or adults."					
Camfield et al 1992	uP	3.2.7.5 page 98	Yes. "The authors reported that following the consumption of aspartame but not of sucrose, the total duration of spike-wave discharge per hour was significantly increased and concluded that aspartame appeared to exacerbate the amount of EEG spike wave in children with absence seizures."		N=10 children with absence seizures. "The Panel noted that the combination of the two parameters (number and length of spike-wave bursts) into a single measure was not adequately explained, and lack of control of food and drink intake before and after dosing may have affected the results. The Panel further noted that aspartame was given in a single dose at the ADI." But other studies, with negative findings that used single doses, were treated by the panel as if reliable (ie as uN), and it is not as if the chosen indicators used for negative studies have always been adequately explained.
Shaywitz et al 1994b	rN	3.2.7.5 page 99		"...there was no difference in the occurrence of seizures between aspartame and placebo exposure."	n=10 children with epilepsy
Rowan et al 1995	rN	3.2.7.5 page 99		"The authors reported no seizures or other adverse effects from aspartame ingestion."	n=18
"The Panel noted that the changes in the plasma phenylalanine levels in the studies described above (Shaywitz et al, 1994b; Rowan et al, 1995) were consistent with those in the toxico-kinetic studies. Overall the Panel concluded that the available data did not provide evidence for a relationship between aspartame consumption and seizures." (p 99)					

Schiffman et al 1987	rN	3.2.7.6 page 99		"The authors reported that the incidence rate of headache after consumption of aspartame (35%) was not significantly different from that after placebo (45%)."	N=40. **The ANS panel failed to acknowledge any of the published criticisms of the Schiffman et al study, eg Lipton et al, 'Aspartame and Headache', *NEJM*, 5 May 1988, p 1200; Steinmetzer et al, *NEJM*, 5 May 1988, p 1201; Elias, NEJM, 5 May 1988, p 1201; Edmeads' 'Aspartame and Headache', *Headache, Feb 1988, pp 64-5;* or the limitations of the response from Schiffman et al, *NEJM*, 5 May 1988, p 1201-2. One bizarre feature of those comments from Schiffman et al is on page 1201, where they argued against others that "..aspartame is never consumed in its pure form..." but then on the following page insist that "The use of aspartame capsules in clinical studies is appropriate...", thereby undermining either their defence against their critics, or their own study, both.**
Koehler and Glaros 1988	uP	3.2.7.6 page 99, 101	Yes, "...a significant increase in the frequency of migraine headaches from the placebo to the aspartame treatment (mean number of migraines per subject: 1.72 (baseline phase), 1.55 (placebo phase), and 3.55 (aspartame phase)."		N=11 (not =10 as the panel mistakenly asserted) - 2 males and 9 females (rather than the 8 reported by ANS panel p 99, Sec 3.2.7.6. para 2) "The Panel noted that the high inter-individual variability in the response of the remaining volunteers makes interpretation unreliable."
Lipton et al 1989	uP	3.2.7.6 page 99, 101	Yes. "About 8% reported aspartame as a trigger of headaches compared to 2.3% for carbohydrates, and to about 50% for alcohol."		N=171 The ANS document stated: "The Panel considered that having only listed possible triggers of headaches, was a major limitation of this study." **But that comment is bizarre. If the authors had listed all impossible or unlikely triggers, would that limitation have been overcome? The panel failed to explain itself clearly.**
Van den Eeden et al 1994	uP	3.2.7.6 page 100, 101	Yes. "The authors concluded that a small subset of the population may be susceptible to headaches induced by aspartame."		N=32, high number of dropouts because of adverse effects

“Overall, the panel noted that because of the limitations of the studies it is not possible to conclude on a relationship between aspartame consumption and headaches” Then on p101 "Although the results of a questionnaire-based study (Lipton et al., 1989) and two double-blind out-patient investigations (Koehler and Glaros, 1988; Van den Eeden et al., 1994) employing daily doses of up to 30 mg/kg bw/day indicated a potential association between aspartame intakes and headache, **it is still not possible to deduce causality**, as the effect of diet has not been adequately controlled for and the interpretation of the data was complicated by a high dropout rate and a limited experimental design." (emphasis added)					
Szucs et al 1986	rN	3.2.7.8 page 100		Aspartame "…did not affect IgE-mediated histamine release from mast cells in vitro." "Aspartame did not stimulate mast cell or basophil in vivo as assessed by skin testing."	Mast cell proliferation and IgE mediated histamine release
Kulczycki 1986	uP	3.2.7.8 page 100	Yes, "...a case of aspartame induced urticaria confirmed by double blind challenge."		Case report of aspartame induced urticaria. Discounted all self-reported allergic-like reactions because SCF discounted them in 2002 on the grounds that they were not confirmed double blind. But on p 100 the ANS Panel acknowledged that : “Kulczycki (1986) reported a case of **aspartame induced urticaria confirmed by double blind challenge**” (emphasis added) and"..."the Panel cannot exclude the possibility that in rare instances individuals could be susceptible to allergic reactions following aspartame ingestion.” **The panel was unclear as to whether, or not, that evidence was demed reliable. The panel's reasoning remains opaque**
Garriga et al 1991	rN	3.2.7.8 page 100		"The authors concluded that subjects who believed themselves to be allergic to aspartame did not have reproducible reactions."	n=12

Geha et al 1993	rN	3.2.7.8 page 100		"The authors concluded that aspartame and its conversion products were no more likely than placebo to cause allergic symptoms in subjects with a history consistent with hypersensitivity to aspartame."	ANS Panel reported p101 that Kulczycki subsequently commented in 1995: "...that in the Geha et al. (1993) study, several aspects in subject recruitment method, convenience, compensation and safety for subjects, as well as inclusion and exclusion criteria and challenge may limit the conclusions of their study." "Because of these deficiencies in study design, I am concerned that the NutraSweet Company-sponsored study by Geha et al. does not accurately reflect the incidence of aspartame-induced hives." **But the ANS panel did not comment further on Kulczycki's criticism of this study, which is a puzzling omission. There is no evidence that the panel accepted Kulczucki's complaint or discounted the findings on this study**.
Butchko et al 2002 Review	uP	3.2.7.8 page 101		"When all the research on aspartame, including evaluations in both the premarketing and postmarketing periods, is examined as a whole, it is clear that aspartame is safe, and there are no unresolved questions regarding its safety under conditions of intended use." *Reg. Tox & Pharmacol.* , 35, 2002, p S5	Authors, many of whom worked for the Nutrasweet Company, discounted any and all evidence of putative adverse effects from the consumption of aspartame.
Novick 1985	uP	3.2.7.8 page 101	Yes. "Case[s] of granulomatous panniculitis thought to be related to aspartame were reported"		"The SCF (2002) noted that studies on allergic-like reactions in […other…] individuals who themselves reported such reactions to aspartame have not confirmed the occurrence when later studied under control conditions." **– so far as the panel was concerned, they don't count.**
McCauliffe and Poitras 1991	uP	3.2.7.8 page 101	Yes, "Case[s] of granulomatous panniculitis thought to be related to aspartame were reported"		as above

Veien and Lomholt 2012	uP	3.2.7.8 page 101	Yes, "…few cases of presumed systemic allergic dermatitis in patients with contact sensitivity to formaldehyde, apparently caused by the intake of aspartame in artificial sweeteners, have been described. The four patients described in the literature all had eyelid dermatitis (as cited in Veien et al, 2012)."		4 cases – AMNS: "The Panel noted that the studies available were performed on a limited number of participants."(**As if other studies were performed on unlimited numbers.**)
"The panel considered that the weight of evidence does not suggest that aspartame is associated with allergic-type reactions....however...cannot exclude the possibility that in rare instances individuals could be susceptible to allergic reaction following aspartame ingestion."					
Robert 2001, reviewed by EFSA 2010	uP	3.2.7.9 page 101	Yes. "The case reports consisted of reports published in peer reviewed journals and reports compiled by Dr H.J. Roberts and published under the title 'Aspartame Disease – An ignored Epidemic' "The total number of symptoms reported from all sources was 4281, as most cases reported more than one symptom. Headache was the most frequently reported adverse effect (28.5 %), followed by dizziness and giddiness (19.2 %)."		Spontaneous case reports. Panel commented that: "The case reports consisted of reports published in peer reviewed journals and reports compiled by Dr H.J. Roberts and published under the title *Aspartame Disease – An ignored Epidemic* ...The total number of symptoms reported from all sources was 4281, as most cases reported more than one symptom." The Panel said that: "...the number of cases is low when compared with the widespread use and that the effects were mild to moderate." **The panel discounts the evidence in part because 'too few' cases had been reported, but provided no indication of how many cases might have been deemed sufficient. They repeatedly complained that 'that is not enough', in ways that suggested that no quantity could be sufficient.**
Whilst acknowledging that these data exist, the Panel noted that the data do not meet the pre-specified inclusion/exclusion criteria. To ensure a comprehensive risk assessment the Panel examined the recent assessment of anecdotal reports by the EFSA National Expert Group (EFSA, 2010).					
